# WTAP regulates TRAF6 expression and vascular plasticity-associated phenotypes in experimental cell models

**DOI:** 10.3389/fcell.2026.1773936

**Published:** 2026-10-29

**Authors:** Maoquan Liang, Wei Wang, Wendong Wang

**Affiliations:** 1 Department of Interventional Therapy, The First Dongguan Affiliated Hospital, Guangdong Medical University, Dongguan, China; 2 Department of Health Management, The First Dongguan Affiliated Hospital, Guangdong Medical University, Dongguan, China

**Keywords:** hepatocellular carcinoma, m6A modification, TRAF6, vasculogenic mimicry, WTAP

## Abstract

**Background:**

Hepatocellular carcinoma (HCC) is a highly vascular malignancy characterized by marked tumor vascular plasticity. Vasculogenic mimicry (VM)-associated phenotypes have been linked to aggressive tumor behavior and reduced sensitivity to antiangiogenic therapy, but the epitranscriptomic mechanisms contributing to these phenotypes in HCC remain incompletely defined. This study examined whether Wilms tumor 1-associated protein (WTAP) regulates cell growth and vascular plasticity-associated phenotypes in experimental models through m6A-associated post-transcriptional regulation of TNF receptor-associated factor 6 (TRAF6).

**Methods:**

WTAP and TRAF6 expression was measured in HepG2 and BEL-7402 cells and the hepatocyte comparator using RT-qPCR and Western blotting. Gain- and loss-of-function experiments were performed to assess cell viability, wound closure, invasion, the ability of the examined cells to support vascular-like network formation in tumor cell-HUVEC co-culture, and the expression of vascular plasticity- and EMT-associated proteins. MeRIP-qPCR was used to evaluate m6A enrichment of TRAF6 transcripts, and an Actinomycin D chase assay was performed to assess TRAF6 mRNA stability. WT/MUT reporter assays, YTHDF1 RNA immunoprecipitation followed by qPCR (RIP-qPCR), and YTHDF1 depletion were used to examine additional aspects of TRAF6 regulation. Subcutaneous xenograft models were used to evaluate tumor growth and PAS-positive/CD31-negative VM-like structures.

**Results:**

WTAP and TRAF6 were upregulated in the examined cells. WTAP overexpression enhanced cell viability, wound closure, invasion, tumor cell-supported vascular-like network formation, and vascular plasticity/EMT-associated molecular changes. WTAP manipulation altered TRAF6 mRNA abundance, m6A enrichment, and decay; its effect was greater on the WT than the MUT reporter. WTAP overexpression also increased TRAF6 RNA recovery in YTHDF1 immunoprecipitates. YTHDF1 depletion reduced the associated TRAF6 protein increase despite persistently elevated mRNA. TRAF6 knockdown attenuated WTAP-driven phenotypes and tumor growth, while TRAF6 overexpression partially rescued the effects of WTAP knockdown. PAS/CD31 staining provided qualitative evidence of VM-like morphology.

**Conclusion:**

WTAP contributes to the observed cellular and xenograft phenotypes, partly through TRAF6 post-transcriptional regulation associated with altered m6A enrichment and mRNA stability. YTHDF1 may regulate TRAF6 protein, but its role in mRNA stabilization remains unproven. The precise m6A sites and perfusion of VM-like structures remain unclear and warrant validation in clinically relevant HCC models and human specimens.

## Introduction

Liver cancer remains a major cause of cancer-related mortality worldwide, and hepatocellular carcinoma (HCC) represents its predominant histological subtype. HCC develops through heterogeneous molecular and etiological processes and is characterized by frequent recurrence, metastatic progression, and limited long-term survival in patients with advanced disease (Llovet et al., 2021). Despite advances in molecularly targeted therapy, immune checkpoint blockade, and combination treatment, primary and acquired resistance remain major clinical challenges. HCC is also a highly vascularized malignancy in which angiogenic signaling and dynamic remodeling of the tumor vasculature support tumor growth, dissemination, metabolic adaptation, and treatment resistance (Morse et al., 2019; Yao et al., 2023). Although VEGF/VEGFR-targeted agents have become central components of systemic HCC treatment, their efficacy may be restricted by tumor heterogeneity, adaptive vascular remodeling, and alternative mechanisms of tumor blood supply. Defining the molecular basis of noncanonical vascularization may therefore provide additional insight into HCC progression and resistance to antiangiogenic therapy.

Vasculogenic mimicry (VM) was originally described as the capacity of highly aggressive tumor cells to organize into extracellular matrix-rich, vascular channel-like networks without a conventional endothelial lining (Maniotis et al., 1999). Unlike classical angiogenesis, which depends on endothelial-cell proliferation and sprouting, VM reflects phenotypic plasticity of malignant cells and their interaction with the surrounding extracellular matrix (Folberg and Maniotis, 2004; Marques Dos Reis and Vieira Berti, 2022). In histological studies, periodic acid-Schiff (PAS)-positive and CD31-negative channel-like structures are commonly used as an operational criterion for identifying VM-like structures, including in HCC specimens (Yang et al., 2015). However, PAS-positive/CD31-negative morphology alone does not establish that these structures are functionally perfused or connected to the host circulation. Functional confirmation generally requires complementary evidence from three-dimensional tumor-cell assays, intraluminal blood elements, vascular tracers, or direct perfusion analyses.

VM-associated phenotypes overlap extensively with angiogenesis, extracellular matrix remodeling, epithelial-mesenchymal transition (EMT), stem-like plasticity, and hypoxic adaptation. VE-cadherin, matrix metalloproteinases, VEGFA, angiopoietin-2, and EMT-related proteins are frequently altered during VM-associated remodeling, but these molecules are not specific to VM and also participate in endothelial angiogenesis, invasion, and broader tumor aggressiveness programs (Zhang et al., 2007; Ou et al., 2019). In HCC, hypoxia-regulated signaling, EMT, Hippo pathway activity, and extracellular matrix reorganization have all been implicated in the development of VM-associated phenotypes (Ou et al., 2019; Huang et al., 2015; Qiao et al., 2021). Molecular-marker changes should therefore be interpreted together with morphological and functional evidence rather than being regarded as independent proof of VM formation.

TNF receptor-associated factor 6 (TRAF6) is a signaling adaptor and E3 ubiquitin ligase that integrates signals from Toll-like receptors, interleukin-1 receptors, members of the TNF receptor superfamily, and other inflammatory pathways. TRAF6-dependent K63-linked ubiquitination contributes to the activation of NF-κB, mitogen-activated protein kinase, and related signaling networks that regulate cell survival, inflammation, immune responses, invasion, and tissue remodeling. In HCC, elevated TRAF6 expression has been associated with tumor progression and unfavorable clinical outcomes, while experimental suppression of TRAF6 reduces HCC cell growth and invasive behavior (Wu et al., 2020; Li et al., 2021). TRAF6 can also stabilize oncogenic signaling components, including c-Myc, whereas SPOP-mediated ubiquitination and degradation of TRAF6 suppresses HCC growth and metastasis (Wu et al., 2020; Chang et al., 2025). These findings identify TRAF6 as a biologically plausible effector linking inflammatory signaling, malignant progression, and vascular plasticity-associated programs in HCC.

N6-methyladenosine (m6A) is a prevalent internal modification of eukaryotic mRNA that influences transcript processing, nuclear export, stability, translation, and decay. m6A deposition is mediated by a multicomponent writer complex, removed by demethylases, and interpreted by reader proteins that determine the subsequent fate of modified transcripts. METTL3 and METTL14 form the central heterodimeric core of the canonical m6A methyltransferase complex (Liu et al., 2014). WTAP functions as an essential regulatory subunit that facilitates nuclear localization and recruitment of the writer complex to RNA substrates but is not itself the principal catalytic enzyme (Ping et al., 2014). Consequently, manipulation of WTAP evaluates the contribution of a regulatory component of the m6A machinery and does not establish that the observed effects are independent of METTL3, METTL14, or other m6A regulators.

WTAP was selected for investigation because previous studies have implicated it in HCC progression. WTAP is elevated in HCC tissues and has been reported to promote tumor-cell proliferation and cell-cycle progression through m6A-associated regulation of the HuR-ETS1-p21/p27 pathway (Chen et al., 2019). Additional clinical and bioinformatic analyses have associated higher WTAP expression with unfavorable prognosis, tumor proliferation, and altered immune-cell infiltration in HCC(18). Separately, m6A dysregulation has been linked to VM formation in HCC through modulation of Hippo pathway activity, suggesting that RNA methylation may contribute to vascular plasticity-related programs (Qiao et al., 2021).

TRAF6 is also subject to post-transcriptional regulation by the m6A machinery. In intestinal epithelial cells, the m6A reader YTHDF1 was shown to recognize m6A-modified TRAF6 transcripts and enhance their translation during innate immune activation (Zong et al., 2021). This observation provides a mechanistic rationale for examining an m6A-associated relationship between WTAP and TRAF6. However, evidence obtained in an intestinal immune context cannot establish that YTHDF1 or the same reader-dependent mechanism operates in HCC. Whether WTAP influences TRAF6 transcript abundance, stability, or translation in HCC, and which m6A reader proteins participate in this process, therefore remain unresolved.

More recently, WTAP-mediated m6A regulation of TRAF6 was reported in a polycystic ovary syndrome model, where the pathway was linked to oxidative stress and ferroptosis in granulosa cells (Cai et al., 2026). Accordingly, the WTAP-TRAF6 relationship itself is not unique to HCC. The incremental question addressed in the present study is whether WTAP-associated TRAF6 regulation contributes to vascular plasticity-related phenotypes in liver tumor models, a biological context distinct from the previously reported reproductive and intestinal immune settings.

In the present study, we investigated whether WTAP regulates cellular and vascular plasticity-associated phenotypes in the examined models through TRAF6-dependent regulation. WTAP and TRAF6 expression was measured in HepG2 and BEL-7402 cells, followed by gain- and loss-of-function analyses of cell viability, wound closure, invasion, vascular-like network formation, and vascular plasticity/EMT-associated molecular changes. MeRIP-qPCR and Actinomycin D chase experiments were used to evaluate TRAF6 m6A enrichment and mRNA stability, respectively. WT/MUT reporter assays, YTHDF1 RIP-qPCR, and YTHDF1 depletion were additionally used to examine reporter responsiveness, RNA association, and TRAF6 expression at the transcript and protein levels. Rescue experiments were performed to determine whether TRAF6 functionally contributes to WTAP-associated phenotypes. Tumor cell-HUVEC co-culture was used to assess the capacity of the examined cells to support vascular-like network formation, whereas PAS/CD31 staining was used to identify VM-like histological structures in xenograft tumors. Because the co-culture system does not distinguish tumor-cell-autonomous VM from endothelial-assisted network formation, and functional perfusion was not examined *in vivo*, the resulting vascular phenotypes are interpreted as VM-associated and pro-angiogenic changes rather than definitive evidence of functional VM channels.

## Materials and methods

### TCGA-LIHC data acquisition and bioinformatic analysis

RNA-sequencing expression profiles and corresponding clinical annotations for the TCGA Liver Hepatocellular Carcinoma cohort (TCGA-LIHC) were obtained from the Genomic Data Commons/Xena resource. Primary tumor and solid tissue normal samples were included. Expression values were processed as log2 (TPM +1). When multiple samples of the same tissue type were available from the same patient, one representative sample was retained for patient-level analyses, and duplicate exclusions were recorded.

WTAP and TRAF6 expression levels were compared between primary tumor and normal liver tissues. Their co-expression relationship was evaluated in primary tumor samples using Spearman correlation analysis. A WTAP-TRAF6 axis score was calculated for each primary tumor sample as the mean of the z-standardized expression values of WTAP and TRAF6.

Hallmark angiogenesis, hypoxia, epithelial-mesenchymal transition, and TNFα signaling via NF-κB gene sets were obtained from the Molecular Signatures Database. Gene-set activity was estimated at the individual-sample level using single-sample gene-set enrichment analysis (ssGSEA). A VM-related transcriptional signature was calculated using CDH5, EPHA2, LAMC2, MMP2, MMP9, VEGFA, ANGPT2, HIF1A, and VIM. This score was interpreted as a VM-related transcriptional feature rather than as direct evidence of histological or functional vasculogenic mimicry formation.

Histological grade and pathological stage were extracted from TCGA clinical annotations. Overall survival was defined as the interval from diagnosis to death or last follow-up. For Kaplan-Meier analysis, patients were divided into high- and low-expression groups according to the median WTAP expression. Samples lacking variables required for a specific analysis were excluded from that analysis without imputation. All statistical tests were two-sided. Tumor-normal comparisons were performed using Wilcoxon rank-sum tests, paired tumor-normal analyses using Wilcoxon signed-rank tests, clinicopathological group comparisons using Kruskal–Wallis tests followed by Dunn tests with Benjamini–Hochberg correction, and survival differences using log-rank tests and Cox proportional-hazards models where appropriate.

### Cell culture

HepG2 (IM-H038, IMMOCELL, China), BEL-7402 (JNO-H0152, Jennio Biotech, China), and Huh7 (IM-H040, IMMOCELL, China), a supplier-designated nonmalignant human hepatocyte control (IM-H473, IMMOCELL, China), and human umbilical vein endothelial cells (HUVECs; IM-H205, IMMOCELL, China) were obtained from the indicated suppliers. BEL-7402 cells and nonmalignant human hepatocytes were maintained in RPMI-1640 medium (Gibco, United States), HepG2 cells were cultured in Minimum Essential Medium (MEM; STM-CM-0304, Stem Recell, China), and Huh7 cells were cultured in high-glucose Dulbecco’s modified Eagle’s medium (DMEM). These media were supplemented with 10% fetal bovine serum (FBS; FSD500, Excell Bio, China) and 1% penicillin-streptomycin. HUVECs were cultured in endothelial cell medium supplemented with endothelial cell growth supplements, 10% FBS, and 1% penicillin-streptomycin. All cells were maintained at 37 °C in a humidified incubator containing 5% CO_2_. HepG2 is a hepatoblastoma-derived cell line. BEL-7402 is listed as a contaminated, HeLa-derived line in Cellosaurus; it is therefore not treated as an authenticated adult HCC model here (Cellosaurus BEL-7402, 2025; Cellosaurus Hep-G2, 2026).

### RNA extraction and RT-qPCR

Total RNA was isolated using a cell/tissue total RNA isolation kit (V2, RC112, Vazyme, China). RNA concentration and purity were assessed using a NanoDrop spectrophotometer (Nano600, Shanghai Jiapeng Technology Co., Ltd., China). cDNA was synthesized using a HiScript III 1st Strand cDNA Synthesis Kit (R312, Vazyme, China). RT-qPCR was performed using Taq Pro Universal SYBR qPCR Master Mix (Q712, Vazyme, China) on a Bio-Rad CFX96 Touch real-time PCR system (Hercules, CA, United States). Relative transcript levels were calculated using the 2^−ΔΔCt^ method with GAPDH as the reference gene. The WTAP, TRAF6, and GAPDH primer sequences are listed in Table 1. RT-qPCR was also used to assess YTHDF1 knockdown and TRAF6 mRNA expression in the YTHDF1-depletion experiments.

**TABLE 1:** Primer sequences (Sangon Biotech, Shanghai, China).

Primers	Sequence (5’→3′)	
WTAP	Forward	CCA​ACG​AAG​AAC​CTC​TTC​CCA
	Reverse	TGC​AGA​CTC​CTG​CTG​TTG​TT
TRAF6	Forward	GCG​CAC​TAG​AAC​GAG​CAA​G
	Reverse	TTT​CCA​GGG​GTG​GGT​CAA​AC
GAPDH	Forward	AAT​GGG​CAG​CCG​TTA​GGA​AA
	Reverse	GCG​CCC​AAT​ACG​ACC​AAA​TC

## Cell transduction and establishment of stable cell lines

Lentiviral vectors carrying short hairpin RNAs targeting human WTAP (sh-WTAP) or TRAF6 (sh-TRAF6), together with the corresponding nontargeting control vector (sh-NC), were used to establish stable knockdown cell lines. Lentiviral vectors expressing full-length human WTAP (oe-WTAP) or TRAF6 (oe-TRAF6), as well as the corresponding empty-vector control (oe-NC), were used to establish stable overexpression cell lines. HepG2, BEL-7402, and Huh7 cells were seeded in culture plates and cultured to approximately 60%–70% confluence. The cells were then transduced with the indicated lentiviral particles at a lentiviral suspension-to-medium ratio of 1:100 (v/v). No polybrene or other transduction-enhancing reagent was used. After 24 h, the virus-containing medium was replaced with fresh complete culture medium. At 48 h after transduction, cells were selected with 5 μg/mL puromycin until the corresponding nontransduced control cells were completely eliminated. The surviving puromycin-resistant cells were expanded as stable polyclonal cell populations. The candidate shRNA constructs targeting WTAP or TRAF6 were initially evaluated. The construct producing the greatest reduction in the corresponding target expression was selected for subsequent cellular and xenograft experiments. WTAP and TRAF6 protein levels were additionally examined in the five combined-perturbation groups in HepG2, BEL-7402, and Huh7 cells to document the molecular effects of the rescue constructs.

### YTHDF1 depletion

Three YTHDF1-targeting siRNAs (si-YTHDF1 #1, #2, and #3) were evaluated against a nontargeting siRNA control (si-NC) by RT-qPCR. TRAF6 mRNA and TRAF6 and YTHDF1 protein expression were then examined in the oe-NC + si-NC, oe-WTAP + si-NC, and oe-WTAP + si-YTHDF1 groups.

### Western blotting

Total protein was extracted from cultured cells or xenograft tumor tissues using ice-cold RIPA lysis buffer (Beyotime, Shanghai, China) supplemented with phenylmethylsulfonyl fluoride (PMSF). Protein concentrations were determined using a bicinchoninic acid assay kit (WB6501, NCM Biotech, China). Equal amounts of protein were separated by 5%–12% SDS-PAGE and transferred onto polyvinylidene fluoride membranes (Sigma-Aldrich, United States). After blocking with 5% nonfat milk for 1 h at room temperature, the membranes were incubated with the indicated primary antibodies overnight at 4 °C. The membranes were then washed three times with Tris-buffered saline containing 0.1% Tween-20 and incubated with the appropriate horseradish peroxidase-conjugated secondary antibodies for 1 h at room temperature. Protein bands were visualized using an enhanced chemiluminescence detection system and captured using a JP-K600 imaging system (Shanghai Jiapeng Technology Co., Ltd., China). Band intensities were quantified using ImageJ software (Fiji, NIH, Bethesda, MD, United States) and normalized to GAPDH or β-actin.

The following primary antibodies were used at a dilution of 1:1,000: rabbit monoclonal anti-WTAP antibody (clone EPR18744, ab195380), rabbit monoclonal anti-TRAF6 antibody (clone EP592Y, ab40675), rabbit monoclonal anti-angiopoietin-2 antibody (clone EPR2891 (2), ab155106), rabbit polyclonal anti-VEGFA antibody (ab46154), rabbit monoclonal anti-MMP2 antibody (clone EPR1184, ab92536), rabbit monoclonal anti-N-cadherin antibody (clone EPR1791-4, ab76011), and mouse monoclonal anti-E-cadherin antibody (clone 4A2, ab231303), all purchased from Abcam.

Rabbit polyclonal anti-VE-cadherin antibody (#2158), rabbit monoclonal anti-MMP9 antibody (clone D6O3H, #13667), rabbit monoclonal anti-vimentin antibody (clone D21H3, #5741), and rabbit monoclonal anti-β-actin antibody (clone 13E5, #4970) were purchased from Cell Signaling Technology and used at a dilution of 1:1,000. Rabbit monoclonal anti-GAPDH antibody (clone 14C10, #2118; Cell Signaling Technology) was used at a dilution of 1:10,000.

Horseradish peroxidase-conjugated goat anti-rabbit IgG (ab6721, Abcam) and goat anti-mouse IgG (ab6789, Abcam) were used as secondary antibodies at a dilution of 1:20,000.

### Actinomycin D chase assay

To evaluate TRAF6 mRNA stability, HepG2 and BEL-7402 cells carrying stable WTAP knockdown, WTAP overexpression, or the corresponding control vectors were treated with Actinomycin D at a final concentration of 5 μg/mL to inhibit *de novo* transcription. Cells were collected at 0, 3, 6, and 9 h after treatment. Total RNA was extracted and subjected to RT-qPCR analysis of TRAF6 mRNA expression. TRAF6 expression was normalized to GAPDH, and the remaining TRAF6 mRNA level at each time point was expressed relative to the corresponding value at 0 h, which was set to 1. Each experiment was performed independently three times.

### Cell counting kit-8 (CCK-8) assay

Cell viability was assessed using a CCK-8 assay (Bioss, China). We placed 2 × 10^3^ cells per well in a 96-well plate, replicated six times, and incubated them for 6 h at 37 °C and 5% CO_2_. After the indicated incubation periods in a CO_2_ incubator, the absorbance at 450 nm was detected by a microplate reader (Multiskan FC 357–714018, Thermo Scientific, United States).

### Wound healing assays

After scratching and washing away detached cells, the cells were cultured in serum-free medium during the 24-h wound-healing period to reduce the potential contribution of proliferation to wound closure. In six-well plates, we grew the cells until they reached confluence (1 × 10^6^ cells/mL). A straight scratch was made across the cell monolayer in each well using a 1-mL pipette tip for cell wounding. Images at the regions where the scratch line intersected with the grid markings were acquired at 0 and 24 h during incubation. We utilized ImageJ software (Fiji, NIH, Bethesda, Maryland, United States) to measure the regions. Relative wound closure rates (%) were calculated using this equation.

### Transwell assays

Cells from the indicated treatment groups were resuspended in serum-free medium and seeded into the upper chambers of Matrigel-coated Transwell inserts at a density of 2 × 10^5^ cells/mL. The lower chambers were filled with 600 μL of complete culture medium containing 10% FBS as a chemoattractant. After incubation at 37 °C for 24 h, non-invading cells remaining on the upper surface of the membrane were gently removed with a cotton swab. Cells that had invaded through the Matrigel-coated membrane were fixed with 4% paraformaldehyde and stained with crystal violet. Representative fields were photographed using a ZEISS microscope, and the number of invaded cells was quantified using ImageJ software. Each experiment was performed independently three times.

### Tumor cell-HUVEC vascular-like network formation assay

HUVECs were co-cultured with HepG2 or Huh7 cells carrying the indicated stable WTAP or TRAF6 modifications to evaluate the ability of the examined cells to support vascular-like network formation. Matrigel was thawed at 4 °C overnight, dispensed into pre-chilled 96-well plates, and allowed to polymerize at 37 °C for 30 min. HUVECs and the examined cells were mixed at a ratio of 1:1 and resuspended at a total cell density of 2 × 10^5 cells/mL. The mixed cell suspension was seeded onto the Matrigel-coated wells and incubated at 37 °C for 16 h. Vascular-like networks were then photographed using an inverted microscope. The number of nodes, junctions, and meshes, together with total network length, was quantified using the Angiogenesis Analyzer plugin in ImageJ. Each experiment was performed independently three times.

### Methylated RNA immunoprecipitation

RNA was fragmented in the fragmentation buffer of an m6A MeRIP kit (GenSeq, Shanghai Cloud-seq Biotech Co., China) at 70 °C for 5 min, cooled, and purified. Anti-m6A antibody was incubated with magnetic beads for 1 h at room temperature. Fragmented RNA was then incubated with the antibody-bead complex at 4 °C with gentle rotation for approximately 1 h. After washing to remove nonspecifically bound RNA, the bound RNA was eluted and purified for RT-qPCR analysis. Input RNA was retained as a control.

The MeRIP-qPCR assay was interpreted as a transcript-level measure of relative TRAF6 m6A enrichment among the indicated conditions. It was not used to infer direct physical binding of WTAP to TRAF6 RNA or to map methylation to an individual adenosine.

### WT/MUT dual-luciferase reporter assay

The relative luciferase readout was compared among WT + oe-NC, WT + oe-WTAP, MUT + oe-NC, and MUT + oe-WTAP reporter conditions to assess whether the WTAP-associated response differed between the WT and MUT constructs.

### YTHDF1 RNA immunoprecipitation

RNA immunoprecipitation was performed using an anti-YTHDF1 antibody or an IgG control in the oe-NC and oe-WTAP groups. TRAF6 RNA recovered in the immunoprecipitated fractions was evaluated by RT-qPCR to assess its association with YTHDF1.

### Xenograft tumor models

Male nude mice aged 6–8 weeks were purchased from Jiangxi Zhonghong Boyuan Biotechnology Co., Ltd (Animal License No. SYXK-2020–0001) and housed under specific pathogen-free conditions. For the five-group xenograft experiment shown in Figure 1, the mice were randomly divided into the sh-NC + oe-NC, oe-WTAP + sh-NC, oe-WTAP + sh-TRAF6, sh-WTAP + oe-NC, and sh-WTAP + oe-TRAF6 groups, with six mice in each group (n = 6 per group). For the xenograft-tissue Western blot analyses shown in Figure 2, tumor tissues from these five groups were analyzed; the cohort size and the number of tumors used for protein quantification are distinguished in the figure legends. An additional three-group xenograft experiment was performed for Figure 3, including the sh-NC + oe-NC, oe-WTAP + sh-NC, and oe-WTAP + sh-TRAF6 groups, with three mice in each group (n = 3 per group). For tumor cell inoculation, mice were anesthetized by intraperitoneal injection of pentobarbital sodium at 60 mg/kg, and the left flank was disinfected with iodophor. HepG2 cells subjected to the indicated transfections were then subcutaneously injected into the left flank of the mice. Tumor volume was measured every 5 days. At the experimental endpoint, mice were euthanized by gradual exposure to 100% CO_2_ delivered at a displacement rate of 50% of the chamber volume per minute. Death was confirmed by the absence of spontaneous respiration and heartbeat before tumor tissues were collected for subsequent analyses. All animal experiments were approved by the Laboratory Animal Ethics Committee of the First Dongguan Affiliated Hospital of Guangdong Medical University and were conducted in accordance with institutional guidelines and the ARRIVE guidelines. Both xenograft experiments used male mice; sex-dependent effects were not evaluated. The five-group and three-group experiments are reported as separate cohorts and are not pooled.

**FIGURE 1:**
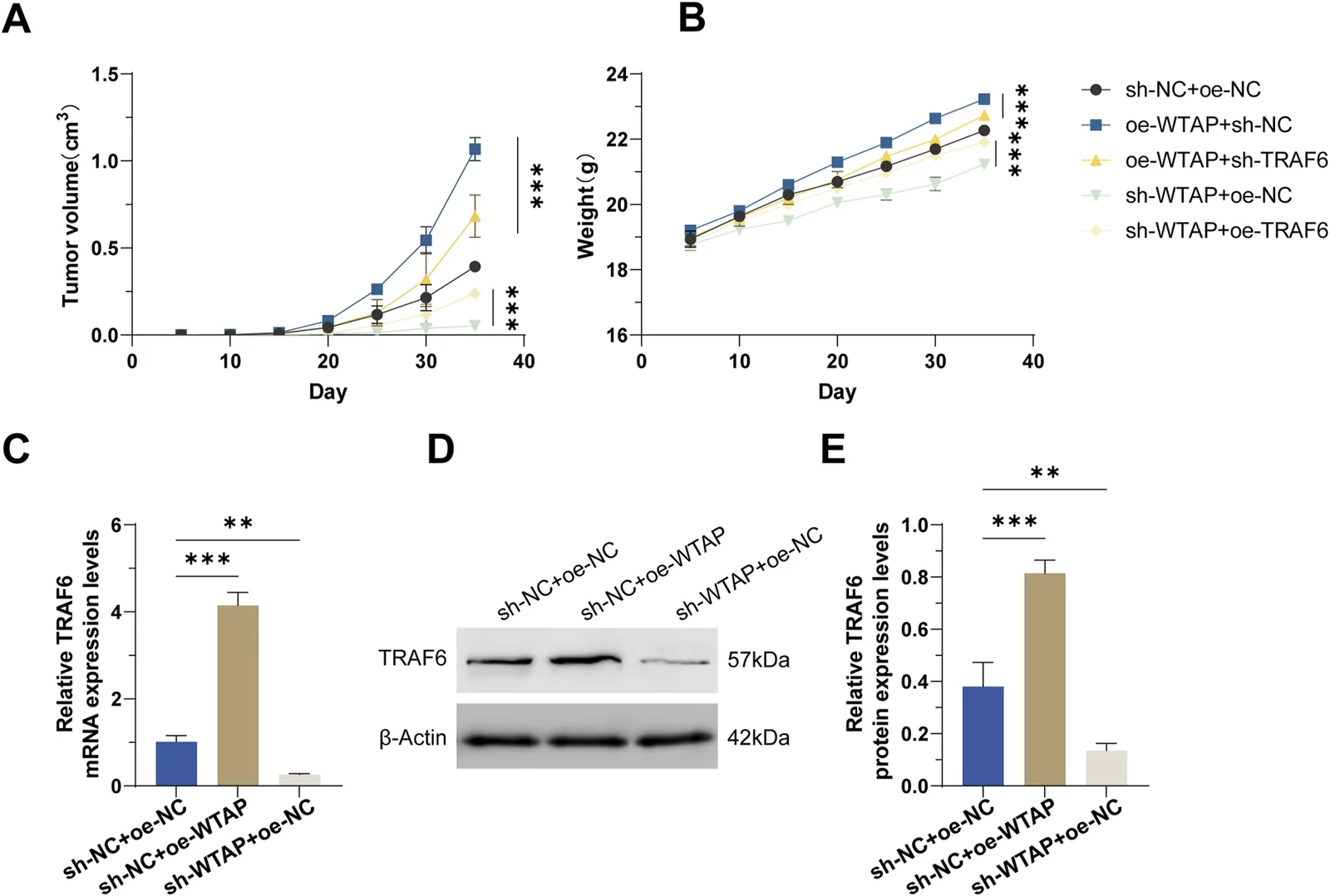
TRAF6 contributes to WTAP-mediated xenograft growth. **(A, B)** Longitudinal changes in tumor volume **(A)** and mouse body weight **(B)** in nude mice inoculated with HepG2 cells carrying sh-NC + oe-NC, oe-WTAP + sh-NC, oe-WTAP + sh-TRAF6, sh-WTAP + oe-NC, or sh-WTAP + oe-TRAF6. Displayed measurements were obtained at 5-day intervals from day 5 to day 35. **(C–E)** RT-qPCR analysis of TRAF6 mRNA expression **(C)**, representative Western blot **(D)**, and densitometric quantification of TRAF6 protein expression **(E)** in xenograft tissues from the three groups shown: sh-NC + oe-NC, oe-WTAP + sh-NC, and sh-WTAP + oe-NC. β-Actin was used as the loading control. Data are presented as the mean ± SD; n = 6 mice per group, with each mouse considered one independent biological replicate. *P < 0.05, **P < 0.01, ***P < 0.001.

**FIGURE 2:**
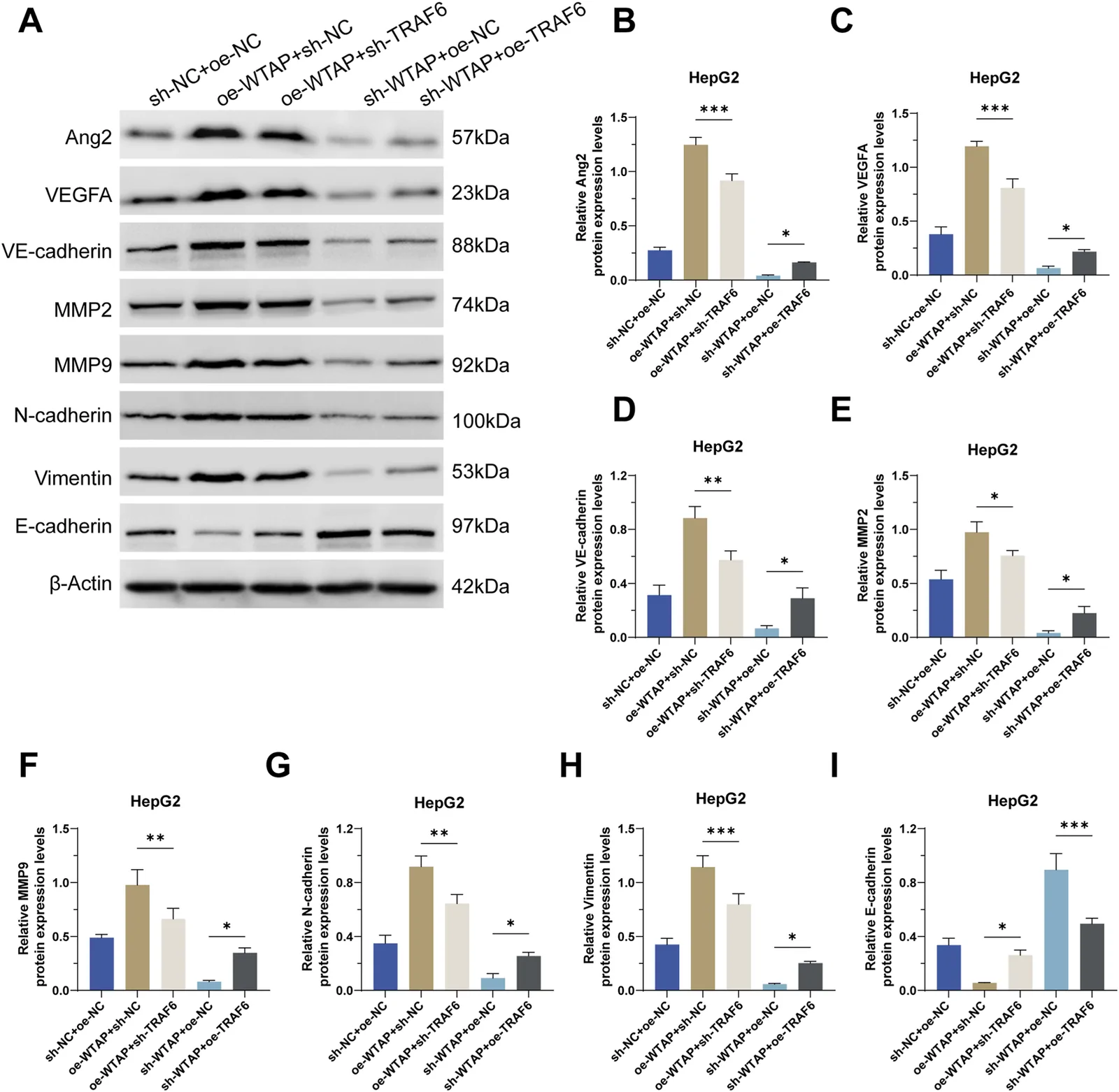
WTAP and TRAF6 regulate vascular plasticity- and EMT-associated protein expression in xenograft tumors. **(A)** Representative Western blots of Ang2, VEGFA, VE-cadherin, MMP2, MMP9, N-cadherin, vimentin, and E-cadherin in xenograft tissues from the indicated groups. β-Actin was used as the loading control. **(B–I)** Densitometric quantification of Ang2 **(B)**, VEGFA **(C)**, VE-cadherin **(D)**, MMP2 **(E)**, MMP9 **(F)**, N-cadherin **(G)**, vimentin **(H)**, and E-cadherin **(I)**. Representative blots are shown, and quantitative data are presented as the mean ± SD from three biologically independent xenograft tumors per group, as stated for this protein analysis. *P < 0.05, **P < 0.01, ***P < 0.001.

**FIGURE 3:**
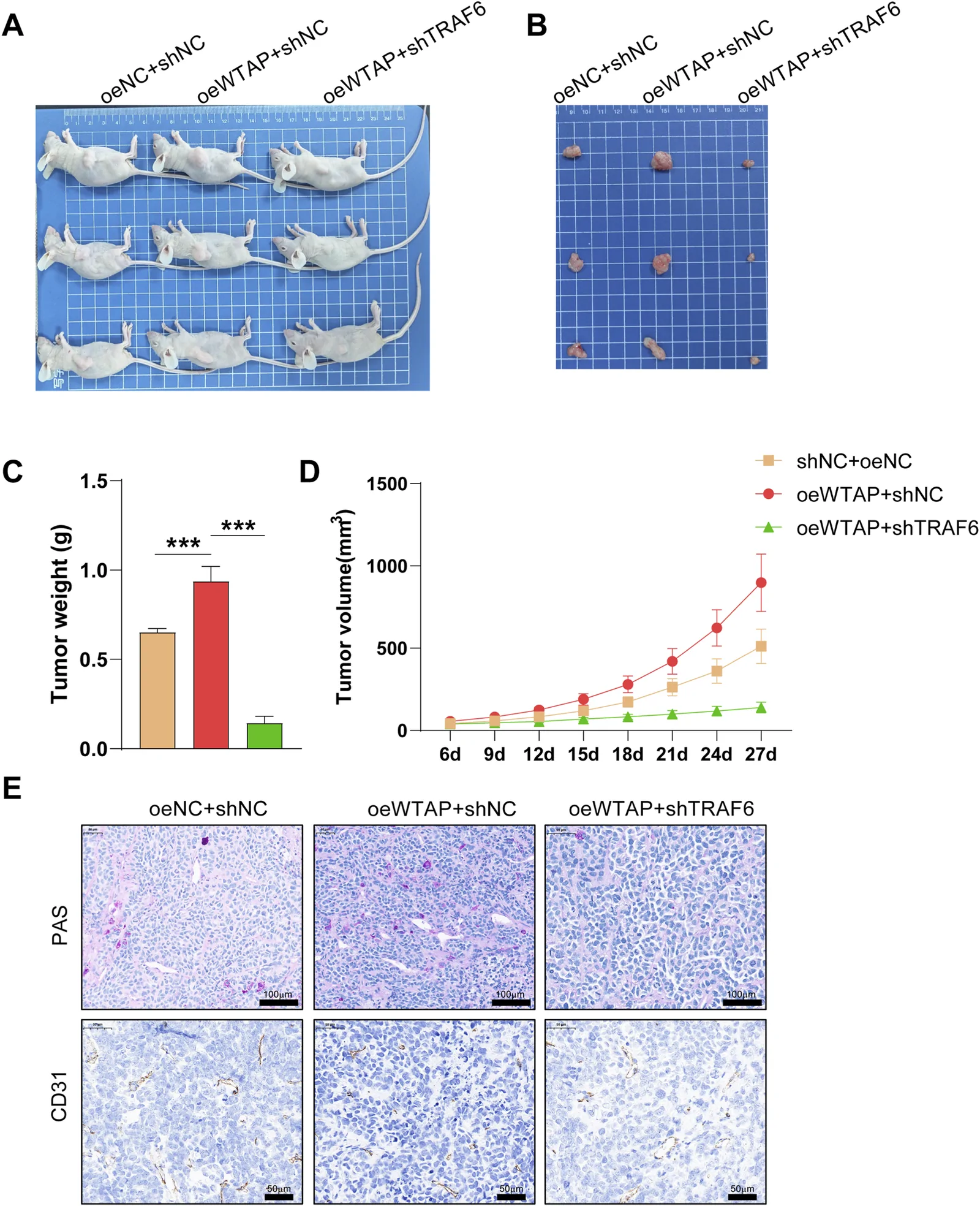
TRAF6 knockdown attenuates WTAP-induced xenograft growth; representative PAS/CD31 histology is shown. **(A)** Representative images of tumor-bearing mice from the sh-NC + oe-NC, oe-WTAP + sh-NC, and oe-WTAP + sh-TRAF6 groups at the experimental endpoint. **(B)** Representative images of excised xenograft tumors. **(C)** Tumor weight in the indicated groups. **(D)** Longitudinal tumor growth curves. **(E)** Representative images of PAS staining and CD31 immunohistochemistry in xenograft tissues for qualitative assessment of VM-like channel morphology. No quantitative VM-like structure count is displayed. Scale bars, 100 μm for PAS and 50 μm for CD31. Quantitative data in C and D are presented as the mean ± SD; n = 3 mice per group, with each mouse considered one independent biological replicate. *P < 0.05, **P < 0.01, ***P < 0.001.

### PAS staining and CD31 immunohistochemistry

Xenograft tumor tissues were fixed in 4% paraformaldehyde, embedded in paraffin, and sectioned. Periodic acid-Schiff (PAS) staining was performed to visualize matrix-rich channel-like structures. CD31 immunohistochemistry was performed on serial tumor sections to distinguish endothelial cell-lined vessels from VM-like structures. PAS-positive/CD31-negative channel-like structures were defined as VM-like structures. Representative images were captured under a light microscope for qualitative assessment of VM-like morphology; no quantitative VM-like structure count is presented in Figure 3E.

PAS and CD31 were evaluated on serial sections rather than by same-section dual staining. The displayed histological assessment was qualitative; no blinded quantitative count of VM-like structures or direct vascular perfusion analysis was performed. The staining is therefore interpreted as supportive of VM-like morphology rather than as definitive evidence of functional VM.

### Statistical analysis

Statistical analyses were performed using GraphPad Prism version 8.0 (GraphPad Software, San Diego, CA, United States). Data are presented as the mean ± standard deviation (SD). The number of independent biological replicates or animals is specified in the corresponding figure legends; technical replicates were not treated as independent observations. Data distribution was assessed before statistical testing. Comparisons between two groups were performed using a two-tailed unpaired Student’s t-test for normally distributed data or the Wilcoxon rank-sum test for non-normally distributed data. Comparisons among multiple groups were performed using one-way analysis of variance followed by Tukey’s multiple-comparisons test. Tumor growth curves were analyzed using two-way repeated-measures analysis of variance with an appropriate *post hoc* multiple-comparisons test. All statistical tests were two-sided, and P < 0.05 was considered statistically significant. Because the PAS/CD31 assessment was qualitative and no VM-like structure count was generated, no inferential statistical test was applied to that histological endpoint.

## Results

### WTAP and TRAF6 expression is elevated in the examined cell lines

To examine the expression patterns of WTAP and TRAF6 in the examined cells, their mRNA and protein levels were compared between nonmalignant human hepatocytes and the examined cell lines HepG2 and BEL-7402. RT-qPCR analysis showed that WTAP and TRAF6 mRNA levels were significantly higher in HepG2 and BEL-7402 cells than in nonmalignant hepatocytes (Figures 4A,B). Consistently, Western blotting demonstrated increased WTAP and TRAF6 protein expression in both cell lines (Figures 4C–E). These findings indicate that WTAP and TRAF6 are concomitantly upregulated in the examined cell models.

**FIGURE 4:**
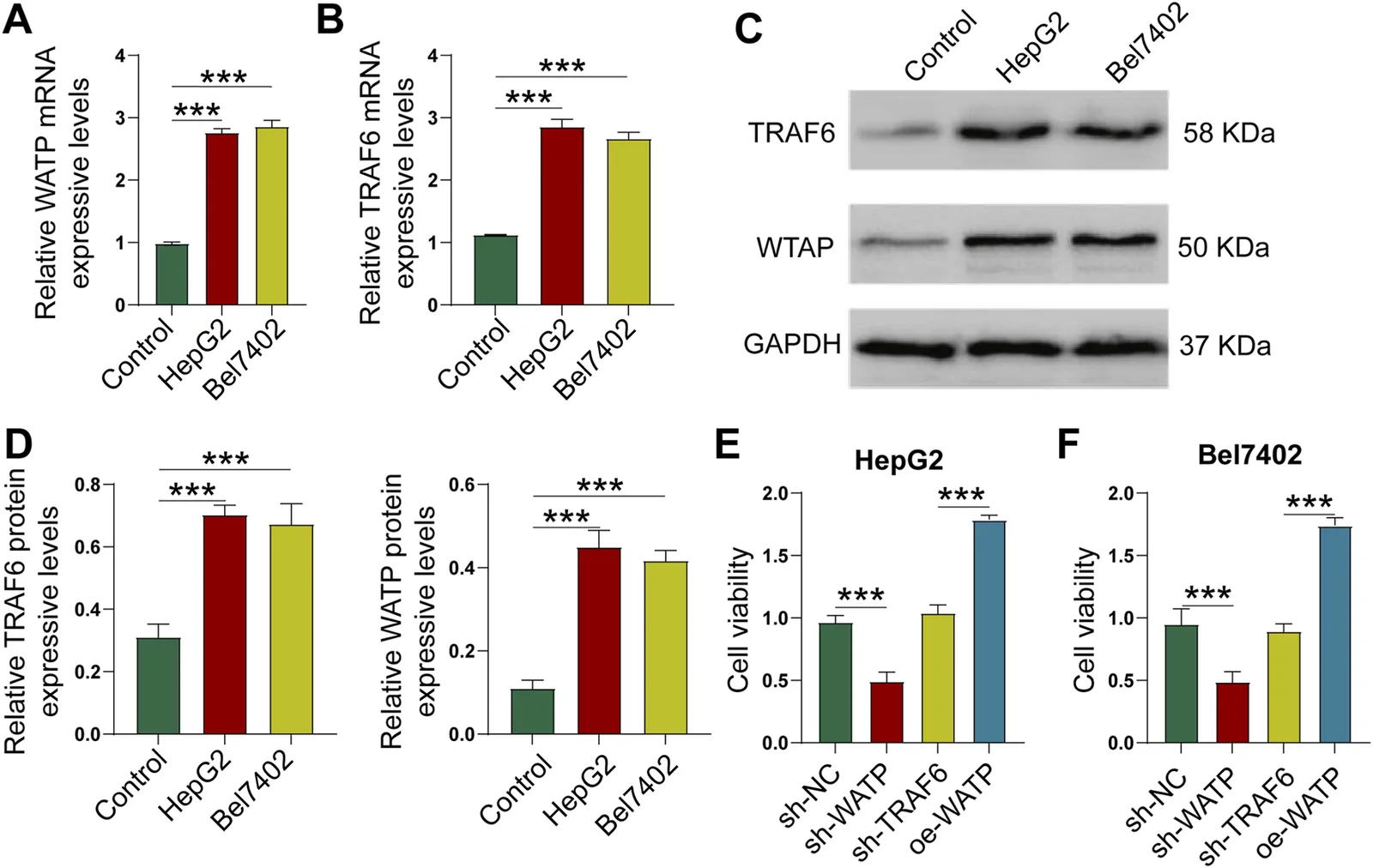
WTAP and TRAF6 expression is elevated in the examined cell lines. **(A, B)** RT-qPCR analysis of WTAP **(A)** and TRAF6 **(B)** mRNA expression in nonmalignant human hepatocytes and the examined cell lines HepG2 and BEL-7402. **(C–E)** Representative Western blots **(C)** and densitometric quantification of TRAF6 **(D)** and WTAP **(E)** protein expression. GAPDH was used as the loading control. **(F, G)** CCK-8 analysis of the viability of HepG2 **(F)** and BEL-7402 **(G)** cells in the sh-NC, sh-WTAP, sh-TRAF6, and oe-WTAP groups shown. Absorbance at 450 nm was measured after 48 h. Data are presented as the mean ± SD from three independent experiments (n = 3). *P < 0.05, **P < 0.01, and ***P < 0.001.

To evaluate the functional contribution of WTAP, HepG2 and BEL-7402 cells with stable WTAP knockdown or overexpression were analyzed using the CCK-8 assay. WTAP knockdown significantly reduced cellular viability, whereas WTAP overexpression increased cellular viability relative to the sh-NC group (Figures 4F,G). These results support a positive role for WTAP in maintaining the viability of the examined cells.

### WTAP expression promoted VM-associated molecular changes in the examined cells

To determine whether WTAP influences molecular programs associated with vascular plasticity and EMT, we examined the expression of angiogenesis-, VM-, and EMT-associated proteins in HepG2 and BEL-7402 cells with stable WTAP knockdown or overexpression.

In HepG2 cells, WTAP knockdown reduced the protein levels of Ang2 and VEGFA, whereas WTAP overexpression increased their expression relative to the corresponding control groups (Figure 5A-C). Similar changes were observed for VE-cadherin, MMP2, and MMP9: their expression was decreased following WTAP knockdown and increased following WTAP overexpression (Figures 5D–F). WTAP knockdown also reduced N-cadherin and vimentin expression while increasing E-cadherin expression. Conversely, WTAP overexpression increased N-cadherin and vimentin levels and decreased E-cadherin expression (Figures 5G–I).

**FIGURE 5:**
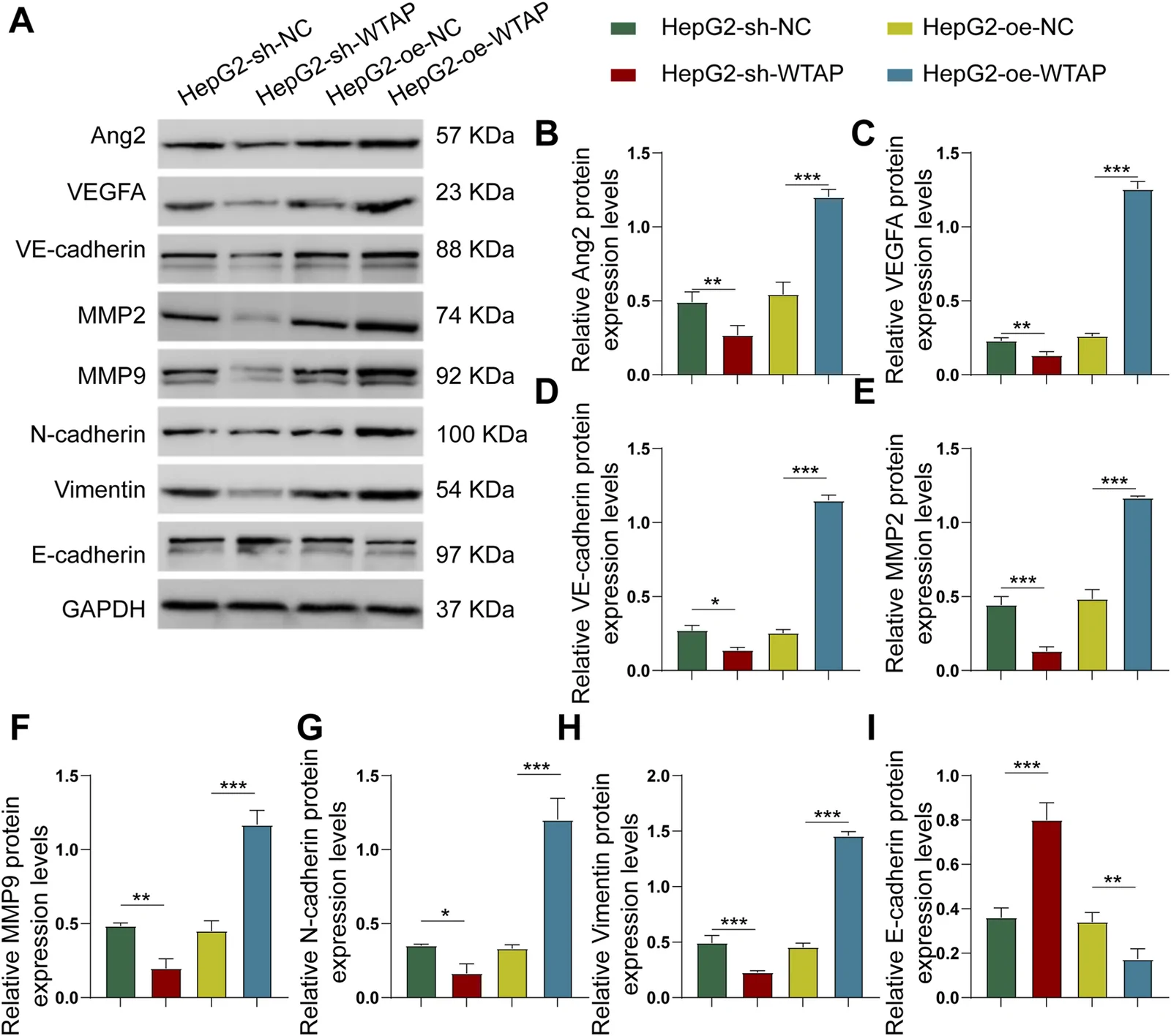
WTAP modulates vascular plasticity- and EMT-associated protein expression in HepG2 cells. **(A)** Representative Western blots of Ang2, VEGFA, VE-cadherin, MMP2, MMP9, N-cadherin, vimentin, and E-cadherin in HepG2 cells carrying sh-NC, sh-WTAP, oe-NC, or oe-WTAP. GAPDH was used as the loading control. **(B–I)** Densitometric quantification of Ang2 **(B)**, VEGFA **(C)**, VE-cadherin **(D)**, MMP2 **(E)**, MMP9 **(F)**, N-cadherin **(G)**, vimentin **(H)**, and E-cadherin **(I)**. Representative blots are shown, and quantitative data are presented as the mean ± SD from three independent experiments (n = 3). *P < 0.05, **P < 0.01, ***P < 0.001.

Comparable expression patterns were observed in BEL-7402 cells. WTAP knockdown decreased Ang2, VEGFA, VE-cadherin, MMP2, MMP9, N-cadherin, and vimentin expression and increased E-cadherin expression, whereas WTAP overexpression produced the opposite effects (Figures 6A–I).

**FIGURE 6:**
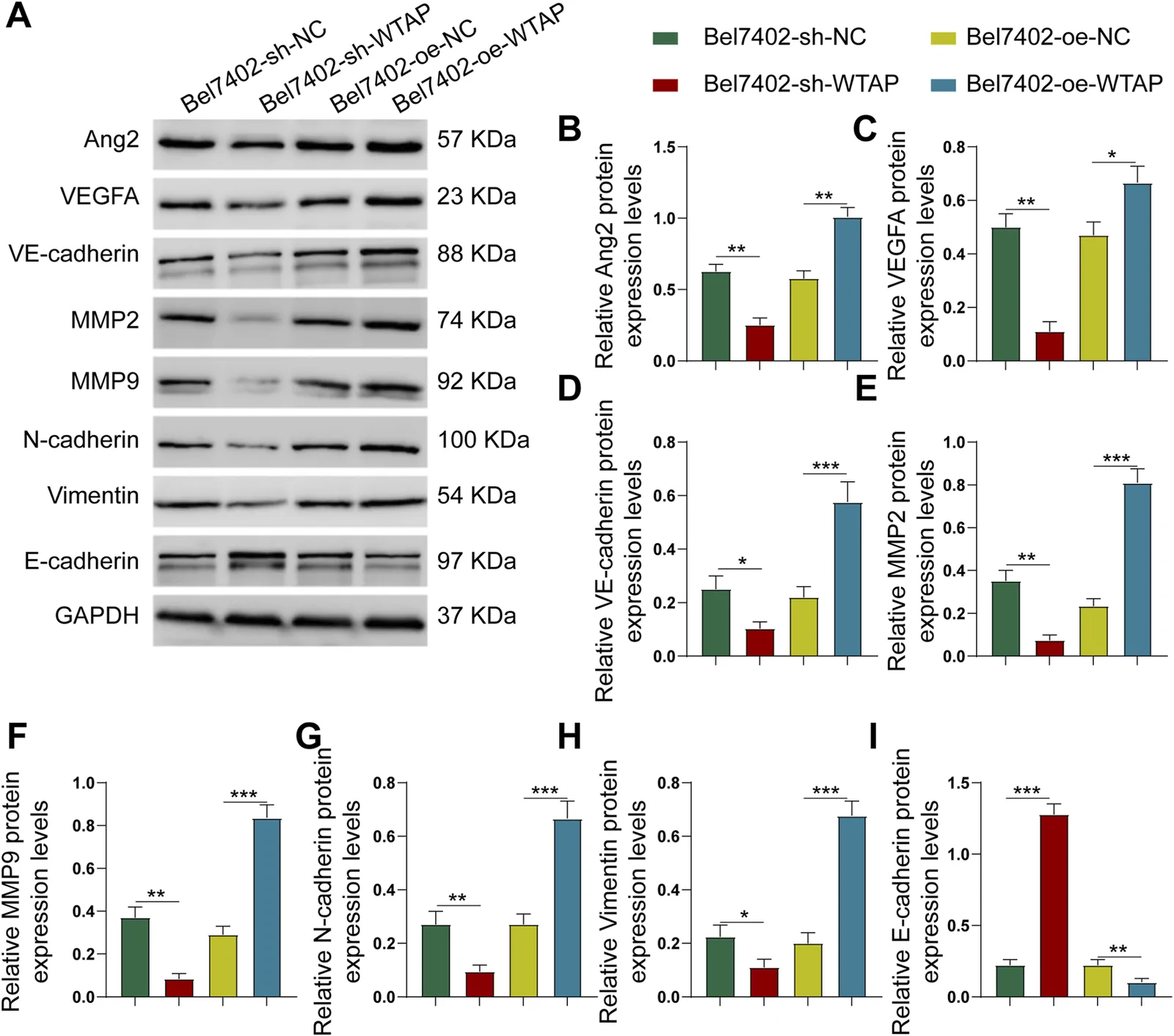
WTAP modulates vascular plasticity- and EMT-associated protein expression in BEL-7402 cells. **(A)** Representative Western blots of Ang2, VEGFA, VE-cadherin, MMP2, MMP9, N-cadherin, vimentin, and E-cadherin in BEL-7402 cells carrying sh-NC, sh-WTAP, oe-NC, or oe-WTAP. GAPDH was used as the loading control. **(B–I)** Densitometric quantification of Ang2 **(B)**, VEGFA **(C)**, VE-cadherin **(D)**, MMP2 **(E)**, MMP9 **(F)**, N-cadherin **(G)**, vimentin **(H)**, and E-cadherin **(I)**. Representative blots are shown, and quantitative data are presented as the mean ± SD from three independent experiments (n = 3). *P < 0.05, **P < 0.01, ***P < 0.001.

Together, these results indicate that WTAP shifts the examined cells toward a protein-expression profile associated with vascular plasticity, extracellular matrix remodeling, pro-angiogenic signaling, and EMT. Because these proteins are not specific markers of VM, the findings are interpreted as evidence of VM-associated and pro-angiogenic molecular changes rather than direct proof of VM formation.

### WTAP enhances tumor cell-supported vascular-like network formation and associated molecular changes

To assess whether WTAP influences vascular network-related phenotypes, HepG2 and Huh7 cells with WTAP or TRAF6 modification were co-cultured with HUVECs on Matrigel. Compared with oe-NC + sh-NC, WTAP overexpression increased the number of junctions and total segment length in both co-culture models. These readouts were lower in the oe-WTAP + sh-TRAF6 group than in the oe-WTAP + sh-NC group (Figures 7A,B). The sh-WTAP + oe-TRAF6 group also showed reduced network formation relative to the control. Because a sh-WTAP + oe-NC group is not included in these panels, this experiment does not independently establish rescue of the network phenotype by TRAF6 overexpression.

**FIGURE 7:**
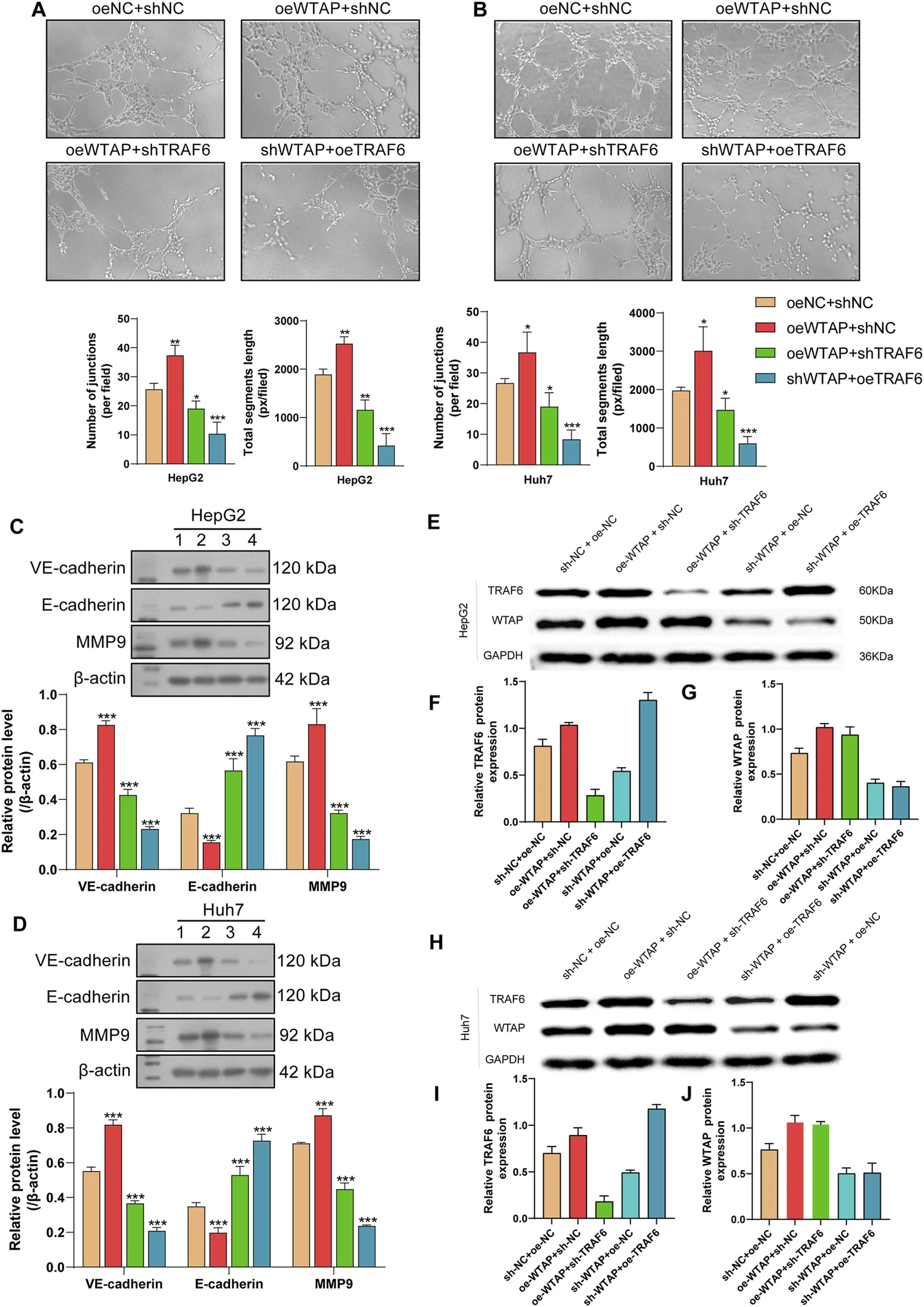
WTAP and TRAF6 perturbation affects tumor cell-supported vascular-like networks and associated proteins. **(A, B)** Representative images and quantification of junction number and total segment length in tumor cell-HUVEC co-cultures containing HepG2 **(A)** or Huh7 **(B)** cells. The four displayed groups are oe-NC + sh-NC, oe-WTAP + sh-NC, oe-WTAP + sh-TRAF6, and sh-WTAP + oe-TRAF6. **(C, D)** Representative Western blots and quantification of VE-cadherin, E-cadherin, and MMP9 in HepG2 **(C)** and Huh7 **(D)** cells; β-actin was used as the loading control. Lanes 1–4 correspond to the four groups above. **(E–G)** Construct-validation blots **(E)** and quantification of TRAF6 **(F)** and WTAP **(G)** in HepG2 cells. **(H–J)** Corresponding blots **(H)** and quantification of TRAF6 **(I)** and WTAP **(J)** in Huh7 cells. For E-J, the five groups are sh-NC + oe-NC, oe-WTAP + sh-NC, oe-WTAP + sh-TRAF6, sh-WTAP + oe-NC, and sh-WTAP + oe-TRAF6; GAPDH was used as the loading control. Panels A–D show mean ± SD from three independent experiments as reported for the original assays. Added quantitative panels F, G, I, and J show three plotted observations per group, with bars and error bars representing mean ± SD. *P < 0.05, **P < 0.01, ***P < 0.001.

Western blotting showed that WTAP overexpression increased VE-cadherin and MMP9 expression while decreasing E-cadherin expression. TRAF6 knockdown attenuated these changes in WTAP-overexpressing cells. The sh-WTAP + oe-TRAF6 group showed lower VE-cadherin and MMP9 and higher E-cadherin levels than the control (Figures 7C,D). Additional Western blots documented WTAP and TRAF6 protein expression across all five construct-validation groups in HepG2 (Figures 7E–G) and Huh7 (Figures 7H–J) cells. TRAF6 protein decreased after sh-TRAF6 treatment in WTAP-overexpressing cells and increased after oe-TRAF6 treatment in WTAP-knockdown cells, while the corresponding WTAP perturbation remained apparent. These validation experiments support interpretation of the combined perturbations but do not supply the missing network-formation comparison. Because the network assay used tumor cell-HUVEC co-culture, it does not establish tumor-cell-autonomous VM formation.

### WTAP-associated TRAF6 RNA regulation is accompanied by a WT/MUT reporter response and YTHDF1-dependent protein regulation

To determine whether WTAP regulates TRAF6 at the post-transcriptional level, TRAF6 mRNA expression was examined in HepG2 and BEL-7402 cells carrying sh-NC + oe-NC, oe-WTAP + sh-NC, or sh-WTAP + oe-NC. RT-qPCR analysis showed that WTAP knockdown significantly reduced TRAF6 mRNA levels, whereas WTAP overexpression increased TRAF6 mRNA expression relative to the corresponding control groups (Figures 8A,B).

**FIGURE 8:**
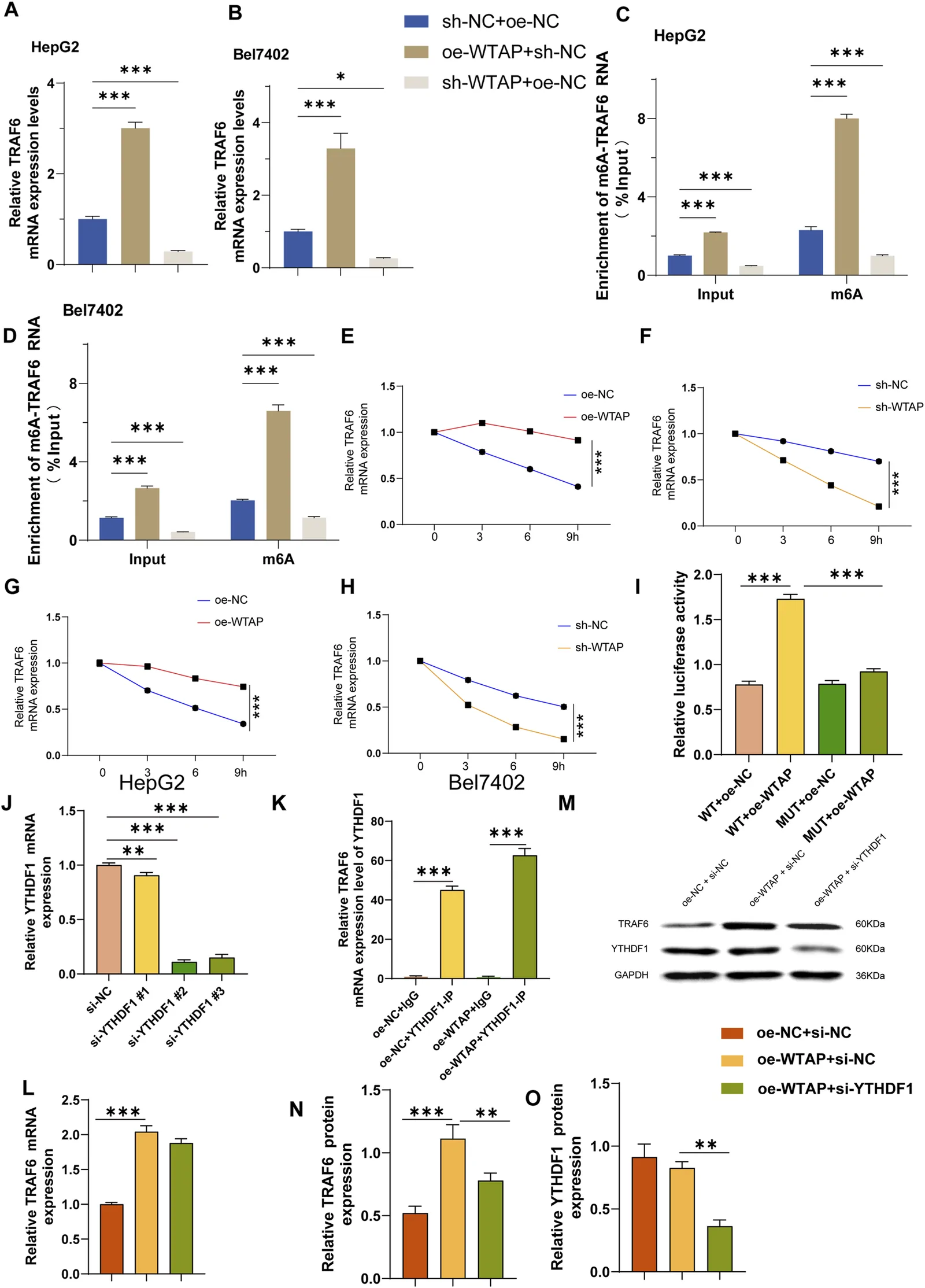
WTAP-associated TRAF6 RNA regulation and evidence for YTHDF1 involvement in TRAF6 protein expression. **(A, B)** RT-qPCR analysis of TRAF6 mRNA in HepG2 **(A)** and BEL-7402 **(B)** cells carrying sh-NC + oe-NC, oe-WTAP + sh-NC, or sh-WTAP + oe-NC. **(C, D)** MeRIP-qPCR analysis of TRAF6 RNA in HepG2 **(C)** and BEL-7402 **(D)** cells from the same groups, with input and anti-m6A fractions displayed. **(E–H)** Actinomycin D chase analysis of TRAF6 mRNA after WTAP overexpression **(E, G)** or knockdown **(F, H)** in HepG2 **(E, F)** and BEL-7402 **(G, H)** cells, normalized to the corresponding 0-h value. **(I)** Relative luciferase readout for WT and MUT reporter constructs with oe-NC or oe-WTAP. **(J)** RT-qPCR assessment of YTHDF1 mRNA after treatment with si-NC or three YTHDF1-targeting siRNAs. **(K)** TRAF6 RNA recovered in IgG or YTHDF1 immunoprecipitates in the oe-NC and oe-WTAP groups. **(L)** RT-qPCR analysis of TRAF6 mRNA in oe-NC + si-NC, oe-WTAP + si-NC, and oe-WTAP + si-YTHDF1 cells. **(M–O)** Representative Western blots (M) and densitometric quantification of TRAF6 **(N)** and YTHDF1 **(O)** in the same three groups; GAPDH was used as the loading control. Panels A–H show mean ± SD from three independent experiments as reported for the original assays. Added quantitative panels I–L, N, and O show three plotted observations per group, with bars and error bars representing mean ± SD. Statistical annotations are retained for the comparisons shown; absence of an annotation does not establish equivalence. *P < 0.05, **P < 0.01, ***P < 0.001.

MeRIP-qPCR was subsequently performed to evaluate m6A enrichment of TRAF6 transcripts. WTAP overexpression increased the recovery of TRAF6 RNA in the anti-m6A immunoprecipitated fraction, whereas WTAP knockdown reduced TRAF6 m6A enrichment in both cell lines (Figures 8C,D). These data indicate that WTAP expression is positively associated with the m6A enrichment of TRAF6 transcripts.

To further assess whether WTAP affects TRAF6 mRNA stability, an Actinomycin D chase assay was performed. Following transcriptional inhibition, WTAP overexpression delayed TRAF6 mRNA decay, whereas WTAP knockdown accelerated TRAF6 mRNA degradation in HepG2 and BEL-7402 cells (Figures 8E–H). Together, these findings support an association between WTAP-dependent changes in TRAF6 m6A enrichment and the maintenance of TRAF6 mRNA stability. These experiments do not identify individual methylated adenosines or establish which reader mediates the change in mRNA stability.

To extend the mechanistic analysis, WT and MUT reporter constructs were compared under control and WTAP-overexpression conditions. WTAP overexpression increased the WT reporter readout, whereas the response of the MUT reporter was smaller. A residual increase remained detectable for MUT (Figure 8I), indicating attenuation rather than complete loss of reporter responsiveness.

YTHDF1 knockdown was first evaluated at the mRNA level. Of the three siRNAs tested, si-YTHDF1 #2 showed the lowest remaining YTHDF1 mRNA signal (Figure 8J). RIP-qPCR detected greater TRAF6 RNA recovery with anti-YTHDF1 than with IgG in both the oe-NC and oe-WTAP groups, and recovery in the YTHDF1 fraction was higher after WTAP overexpression (Figure 8K). These results support an association between YTHDF1 and TRAF6 RNA.

TRAF6 mRNA remained elevated in the oe-WTAP + si-YTHDF1 group (Figure 8L). No statistical comparison between the two WTAP-overexpression groups is annotated in that panel, so equivalence of their mRNA levels cannot be inferred. In contrast, Western blotting showed that YTHDF1 depletion reduced TRAF6 protein relative to oe-WTAP + si-NC and confirmed decreased YTHDF1 protein (Figures 8M–O). Together, these observations implicate YTHDF1 in WTAP-associated TRAF6 protein regulation but do not establish that YTHDF1 mediates TRAF6 mRNA stabilization.

### TRAF6 contributes to WTAP-mediated cell viability, wound closure, and invasion

To determine whether TRAF6 contributes to the cellular effects of WTAP, rescue experiments were performed in HepG2 and BEL-7402 cells with stable WTAP and/or TRAF6 knockdown or overexpression. Protein-level validation of the combined perturbations is shown for HepG2 in Figures 7E–G and BEL-7402 in Figures 9G–I. In both cell lines, TRAF6 overexpression increased TRAF6 protein despite WTAP knockdown, whereas TRAF6 knockdown reduced its expression despite WTAP overexpression. CCK-8 assays showed that WTAP overexpression increased cell viability compared with the sh-NC + oe-NC control group. TRAF6 knockdown significantly attenuated this increase in the oe-WTAP + sh-TRAF6 group compared with the oe-WTAP + sh-NC group. Conversely, TRAF6 overexpression partially restored the reduction in cell viability caused by WTAP knockdown, although viability in the sh-WTAP + oe-TRAF6 group remained lower than that in the control group (Figures 9A,B).

**FIGURE 9:**
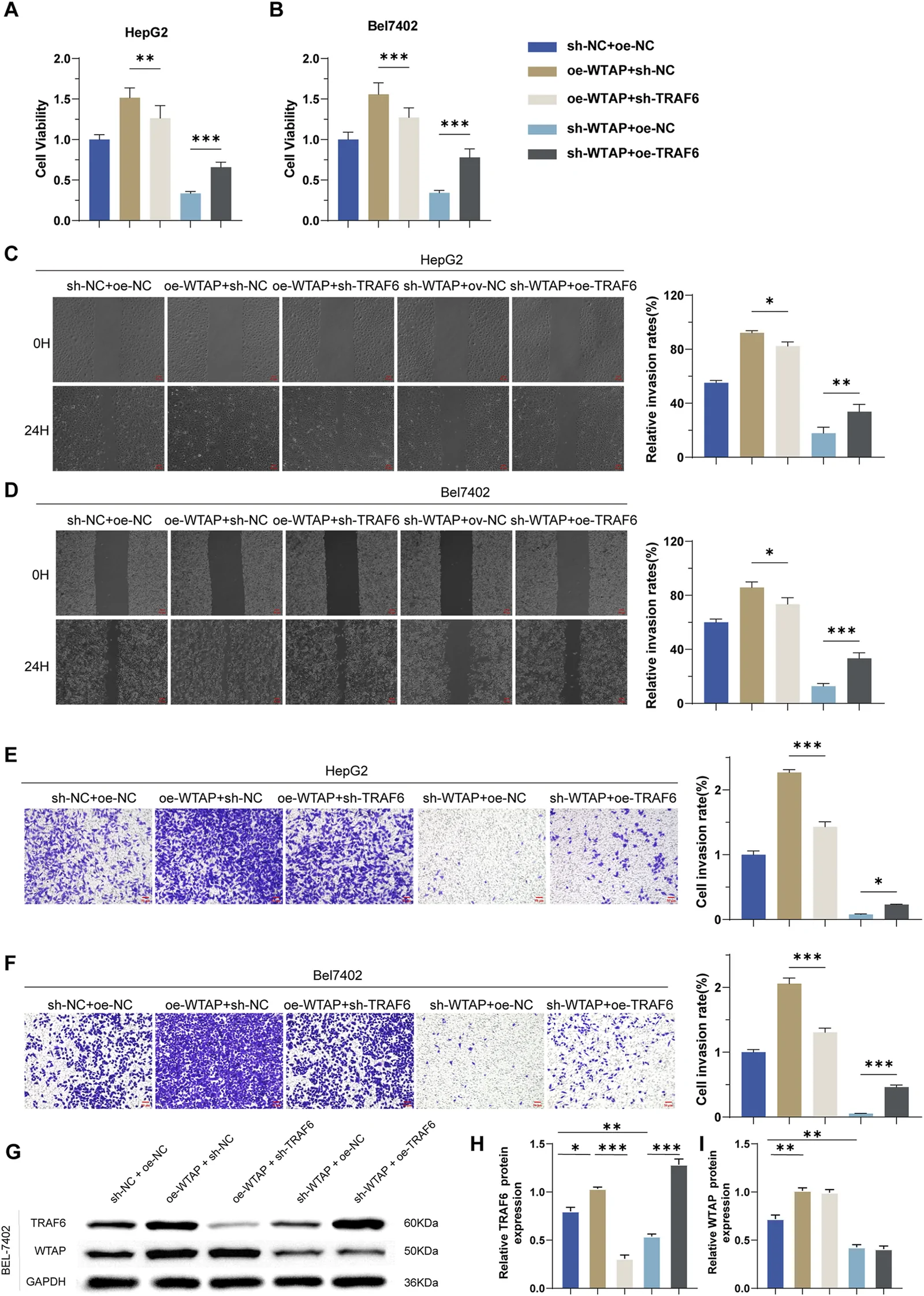
TRAF6 contributes to WTAP-mediated cell viability, wound closure, and invasion. **(A, B)** CCK-8 analysis of the viability of HepG2 **(A)** and BEL-7402 **(B)** cells carrying sh-NC + oe-NC, oe-WTAP + sh-NC, oe-WTAP + sh-TRAF6, sh-WTAP + oe-NC, or sh-WTAP + oe-TRAF6. **(C, D)** Representative images and quantitative analysis of wound closure in HepG2 **(C)** and BEL-7402 **(D)** cells at 0 and 24 h. **(E, F)** Representative images and quantitative analysis of Transwell invasion in HepG2 **(E)** and BEL-7402 **(F)** cells. **(G–I)** Protein-level validation of the five combined-perturbation groups in BEL-7402 cells: representative Western blots **(G)**, TRAF6 quantification **(H)**, and WTAP quantification **(I)**. GAPDH was used as the loading control. Corresponding HepG2 construct validation is shown in Figures 4E–G. Representative images are shown. For A-F, quantitative data are presented as the mean ± SD from three independent experiments (n = 3). For H and I, three observations are plotted per group, and bars and error bars represent mean ± SD. *P < 0.05, **P < 0.01, ***P < 0.001.

Wound-healing assays showed a similar pattern. WTAP overexpression increased wound closure at 24 h, whereas TRAF6 knockdown attenuated the effect of WTAP overexpression. TRAF6 overexpression partially restored wound closure in WTAP-knockdown cells, but the rescue did not return wound closure to the control level (Figures 9C,D).

Transwell invasion assays further showed that WTAP overexpression increased the number of invaded cells in both cell lines. This effect was reduced by TRAF6 knockdown in the oe-WTAP + sh-TRAF6 group. In contrast, TRAF6 overexpression partially rescued the reduction in invasion caused by WTAP knockdown, although the invasive capacity of the sh-WTAP + oe-TRAF6 group remained lower than that of the control group (Figures 9E,F).

Together, these results indicate that TRAF6 contributes to WTAP-mediated regulation of cell viability, wound closure, and invasion. The incomplete rescue observed after TRAF6 overexpression suggests that additional WTAP-regulated factors may also participate in these cellular phenotypes.

### TRAF6 contributes to WTAP-mediated xenograft growth, accompanied by VM-like histological observations

To evaluate the contribution of WTAP and TRAF6 to tumor growth *in vivo*, nude mice were subcutaneously inoculated with HepG2 cells carrying the following stable modifications: sh-NC + oe-NC, oe-WTAP + sh-NC, oe-WTAP + sh-TRAF6, sh-WTAP + oe-NC, or sh-WTAP + oe-TRAF6. Tumor volume was measured at 5-day intervals. WTAP knockdown reduced xenograft growth compared with the control group, whereas TRAF6 overexpression partially restored tumor growth in WTAP-knockdown tumors. However, tumor volume in the sh-WTAP + oe-TRAF6 group remained lower than that in the control group. Conversely, WTAP overexpression increased tumor growth, and this effect was attenuated, but not completely abolished, by TRAF6 knockdown (Figure 1A). Mouse body weight was monitored throughout the experiment and is shown in Figure 1B.

In the three groups shown for xenograft expression analysis, TRAF6 mRNA and protein levels were lower in sh-WTAP + oe-NC tumors and higher in oe-WTAP + sh-NC tumors than in control tumors (Figures 1C–E). These panels support WTAP-associated regulation of TRAF6 expression *in vivo*; they do not show expression measurements for the two TRAF6-rescue groups.

An additional xenograft experiment was performed to further examine whether TRAF6 knockdown attenuated the effects of WTAP overexpression. Nude mice were inoculated with HepG2 cells carrying sh-NC + oe-NC, oe-WTAP + sh-NC, or oe-WTAP + sh-TRAF6. Representative images of tumor-bearing mice and excised tumors showed an increased tumor burden in the oe-WTAP + sh-NC group, whereas TRAF6 knockdown reduced this effect (Figures 3A,B). Consistently, tumor weight and longitudinal tumor growth measurements showed that TRAF6 knockdown attenuated WTAP overexpression-induced xenograft growth (Figures 3C,D).

PAS staining and CD31 immunohistochemistry were used to examine vascular-associated histology in xenograft tissues (Figure 3E). The representative images were used to assess PAS-positive and CD31-negative channel-like morphology, operationally described as VM-like structures. The displayed panels provide qualitative histological evidence; they do not include a quantitative comparison of VM-like structure abundance. Because vascular perfusion was not directly assessed, these images do not establish functional VM channels.

Western blotting of xenograft tissues further showed that WTAP knockdown reduced the expression of Ang2 and VEGFA, whereas TRAF6 overexpression partially restored their expression. Conversely, WTAP overexpression increased Ang2 and VEGFA levels, and this increase was attenuated by TRAF6 knockdown (Figures 9A–C). Similar expression patterns were observed for VE-cadherin, MMP2, and MMP9 (Figures 2D–F). WTAP knockdown also decreased N-cadherin and vimentin expression and increased E-cadherin expression, whereas TRAF6 overexpression partially reversed these changes. In contrast, WTAP overexpression increased N-cadherin and vimentin expression and reduced E-cadherin expression, and TRAF6 knockdown attenuated these effects (Figures 2G–I).

Together, the *in vivo* findings support a contribution of TRAF6 to WTAP-mediated xenograft growth and vascular plasticity- and EMT-associated protein expression, accompanied by qualitative PAS/CD31 histological observations. The incomplete rescue of tumor growth after TRAF6 manipulation suggests that additional WTAP-regulated factors may participate in these processes.

### TCGA-LIHC analysis supports the biological relevance of WTAP and TRAF6 in human HCC

To evaluate the biological relevance of WTAP and TRAF6 in human HCC, we analyzed RNA-sequencing and clinical data from the TCGA-LIHC cohort. WTAP and TRAF6 expression was significantly higher in 371 primary HCC tissues than in 50 solid normal liver tissues (Figures 10A,B). In primary tumor samples, WTAP expression was positively correlated with TRAF6 expression (Figure 10C). A WTAP-TRAF6 axis score was subsequently constructed to evaluate the relationship between their coordinated expression and vascular plasticity-related transcriptional programs. Higher axis scores were positively correlated with a VM-related transcriptional signature and with angiogenesis, hypoxia, EMT, and TNFα/NF-κB pathway scores after Benjamini–Hochberg correction (Figure 10D). WTAP expression showed a modest but statistically significant association with histological grade (Figure 10E). Kaplan-Meier analysis using the median WTAP expression as the predefined cutoff showed that patients with high WTAP expression had shorter overall survival than those with low WTAP expression (Figure 10F). Collectively, these findings provide human HCC transcriptomic and clinical association evidence supporting the biological relevance of WTAP and TRAF6 but do not establish direct m6A regulation, functional VM formation, or independent prognostic value.

**FIGURE 10:**
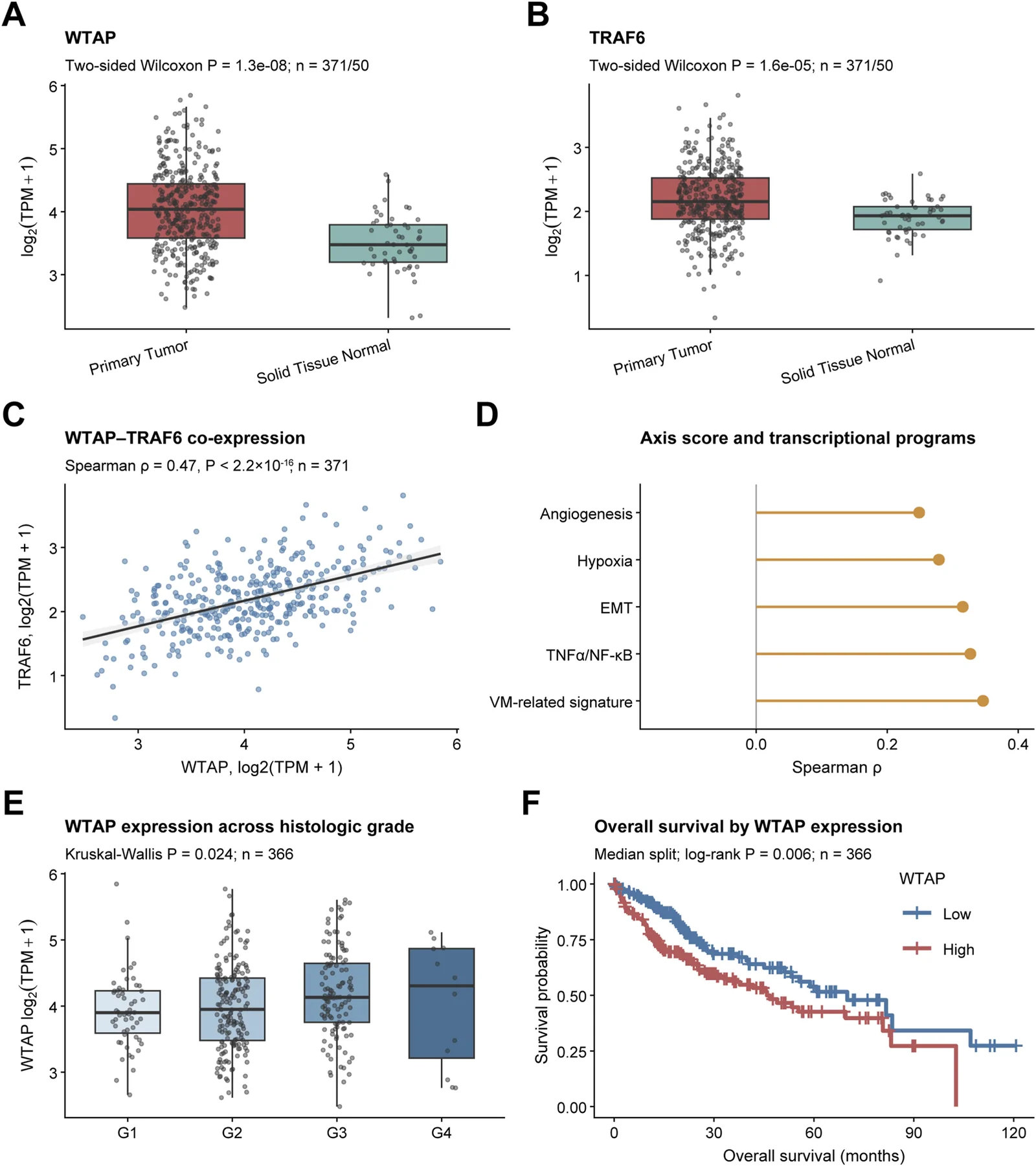
TCGA-LIHC analysis of WTAP, TRAF6, vascular plasticity-related transcriptional programs, and clinical features in HCC. **(A, B)** WTAP **(A)** and TRAF6 **(B)** expression in primary HCC tissues (n = 371) and solid normal liver tissues (n = 50). Differences were evaluated using the two-sided Wilcoxon rank-sum test. **(C)** Spearman correlation between WTAP and TRAF6 expression in primary HCC tissues (n = 371). The fitted line is shown for visualization only. **(D)** Spearman correlations between the WTAP-TRAF6 axis score and angiogenesis, hypoxia, EMT, TNFα/NF-κB, and VM-related transcriptional scores. The axis score was calculated as the mean of z-standardized WTAP and TRAF6 expression. P values were adjusted using the Benjamini–Hochberg method. **(E)** WTAP expression across histological grades in TCGA-LIHC tumors (n = 366). The overall difference was evaluated using the Kruskal–Wallis test. **(F)** Kaplan-Meier analysis of overall survival according to WTAP expression. Patients were divided into WTAP-high and WTAP-low groups using the median expression value as the predefined cutoff (n = 366). Survival curves were compared using the log-rank test.

## Discussion

The present study identifies TRAF6 as a functional contributor to WTAP-associated malignant and vascular plasticity-related phenotypes in the examined models. Stable WTAP knockdown reduced cell viability, wound closure, invasion, and the expression of proteins associated with angiogenic signaling, extracellular matrix remodeling, and EMT, whereas WTAP overexpression produced the opposite effects. WTAP manipulation also altered TRAF6 transcript abundance, m6A enrichment, and mRNA decay kinetics. Rescue experiments showed that TRAF6 knockdown attenuated the effects of WTAP overexpression, while TRAF6 overexpression partially restored the phenotypes suppressed by WTAP knockdown. YTHDF1 RIP and depletion experiments further linked YTHDF1 to TRAF6 RNA association and protein regulation. The tumor-growth findings were supported by xenograft experiments, accompanied by qualitative PAS/CD31 histology. These findings support a WTAP-TRAF6 regulatory relationship in the examined experimental models, although TRAF6 is unlikely to be the only downstream mediator of WTAP.

The tumor-promoting effects of WTAP observed here are consistent with previous HCC studies. WTAP has been reported to promote HCC growth and invasion through m6A- and HuR-dependent regulation of ETS1, and higher WTAP expression has been associated with unfavorable prognosis and aggressive tumor features (Chen et al., 2019; Liang et al., 2022). However, hepatocyte-specific Wtap deficiency was recently shown to promote hepatocarcinogenesis through suppression of proteasome-related genes and activation of GRB2-ERK signaling (Li et al., 2023). This apparent discrepancy may reflect distinct functions of WTAP in nonmalignant hepatocytes during tumor initiation and in established HCC cells during tumor progression. Our findings therefore support a tumor-promoting role for WTAP in the examined cell models rather than a uniform function throughout liver carcinogenesis.

The Actinomycin D chase experiment showed that WTAP overexpression delayed TRAF6 mRNA decay, whereas WTAP knockdown accelerated it. Together with MeRIP-qPCR, these data support an association among WTAP expression, TRAF6 m6A enrichment, and transcript persistence. The additional reporter experiment showed a larger WTAP-associated increase for the WT construct than for the MUT construct, although a smaller increase remained detectable for MUT. Thus, the mutation attenuated rather than abolished reporter responsiveness. YTHDF1 has been shown to recognize m6A-modified TRAF6 transcripts and enhance their translation in intestinal epithelial immune signaling (Zong et al., 2021). Here, TRAF6 RNA was enriched in YTHDF1 immunoprecipitates relative to IgG, and RNA recovery was higher with WTAP overexpression. YTHDF1 depletion also reduced TRAF6 protein in WTAP-overexpressing cells, while TRAF6 mRNA remained elevated. These observations implicate YTHDF1 in TRAF6 protein regulation and are compatible with a contribution at a post-transcriptional step. They do not directly measure translation efficiency, exclude altered protein turnover, or establish that YTHDF1 mediates the WTAP-associated change in TRAF6 mRNA stability. Moreover, increased TRAF6 recovery by RIP could partly reflect increased transcript abundance unless input-normalized occupancy is demonstrated. The evidence therefore supports YTHDF1 involvement without establishing a fully resolved site-specific m6A mechanism.

The novelty of this study should also be interpreted in light of prior disease contexts. WTAP-mediated m6A regulation of TRAF6 has recently been reported in polycystic ovary syndrome (Cai et al., 2026), whereas YTHDF1-dependent TRAF6 regulation was previously described in intestinal epithelial immune signaling (Zong et al., 2021). Thus, the present study does not claim discovery of the WTAP-TRAF6 relationship itself. Rather, it examines whether this regulatory relationship is associated with vascular plasticity-related phenotypes in the examined liver tumor models. In addition, no WTAP RIP/CLIP experiment was performed; therefore, direct physical binding of WTAP to TRAF6 RNA is not established by the current data.

The current reader data are likewise non-exclusive: YTHDF1 is supported as a candidate involved in TRAF6 protein regulation, but YTHDF2, YTHDF3, IGF2BP-family readers, and other m6A-binding proteins were not systematically evaluated and cannot be excluded.

The rescue experiments provide functional support for TRAF6 involvement. TRAF6 knockdown weakened the increases in cell viability, wound closure, invasion, network formation in co-culture, and xenograft growth induced by WTAP overexpression, whereas TRAF6 overexpression partially reversed WTAP-knockdown effects on cell viability, wound closure, invasion, and xenograft growth. The co-culture network experiment did not include the WTAP-knockdown-only comparator required to assess that rescue. The added construct-validation blots strengthen the interpretation of the cellular rescue experiments by documenting WTAP and TRAF6 protein levels in the corresponding groups. This is consistent with recent evidence that SPOP-mediated ubiquitination and degradation of TRAF6 suppresses HCC growth and metastasis (Chang et al., 2025). The incomplete rescue observed across several assays suggests that TRAF6 is one relevant effector within a broader WTAP-regulated RNA network rather than the exclusive mediator of WTAP activity.

Because the rescue was partial, the present design cannot exclude indirect contributions from other WTAP-responsive transcripts or pathways, including previously described WTAP targets in HCC and other tumor contexts. The data therefore support TRAF6 as a functional contributor to WTAP-associated phenotypes, not as the exclusive downstream mediator.

The vascular findings should be interpreted in the context of tumor-cell plasticity. m6A-dependent regulation of YAP1 has previously been linked to VM formation in HCC through the Hippo pathway (Qiao et al., 2021). More recent studies have implicated Twist1-dependent VE-cadherin expression, the Nrf2-ASPM axis, and MAPK/ERK signaling in HCC VM-associated phenotypes (Qin et al., 2024; Zhang Y. et al., 2024; Li et al., 2024). These observations indicate that VM arises from the convergence of hypoxic adaptation, EMT, stem-like plasticity, extracellular matrix remodeling, and endothelial-like differentiation. The coordinated changes in Ang2, VEGFA, VE-cadherin, MMP2, MMP9, E-cadherin, N-cadherin, and vimentin observed here are consistent with this broader phenotype, but none of these proteins is specific for VM.

m6A-dependent regulation of VM is also context dependent across tumor types. For example, METTL3-dependent m6A modification of HOTAIRM1 has been reported to promote VM in glioma (Wu et al., 2023). Together with the METTL3-YAP1/Hippo mechanism reported in HCC(11), these observations indicate that different writers, RNA targets, readers, and downstream pathways may converge on VM-associated phenotypes. The present work differs by focusing on WTAP-associated TRAF6 regulation, but it does not establish a universal or fully resolved m6A-VM pathway.

The tumor cell-HUVEC co-culture assay also limits the interpretation of network formation. Because the examined cells were mixed with endothelial cells on Matrigel, the resulting structures may reflect endothelial organization, tumor-endothelial cooperation, or both. The assay therefore shows that WTAP-modified tumor cells influence vascular-like network formation in the presence of HUVECs but does not demonstrate tumor-cell-autonomous VM. Likewise, PAS-positive/CD31-negative channel-like structures are compatible with VM-like morphology but do not establish functional perfusion or connection to the host circulation. Tumor vascularization may involve overlapping processes, including angiogenesis, vascular co-option, VM, and tumor-endothelial cooperation (Zhang R. et al., 2024). Accordingly, the present findings are described as VM-associated and pro-angiogenic phenotypes rather than definitive functional VM formation.

The TCGA-LIHC analysis provides a human transcriptomic context for the experimental findings. WTAP and TRAF6 were both significantly elevated in primary HCC tissues and showed a moderate positive correlation across tumor samples. Moreover, the WTAP-TRAF6 axis score was positively associated with a VM-related transcriptional signature and with angiogenesis, hypoxia, EMT, and TNFα/NF-κB pathway scores. WTAP expression also differed across histological grades, and high WTAP expression was associated with shorter overall survival in median-based Kaplan-Meier analysis. These observations are consistent with the experimental evidence linking WTAP and TRAF6 to aggressive and vascular plasticity-associated features of HCC.

The clinical analysis therefore addresses part of the requested WTAP-TRAF6-VM-prognosis relationship at the transcriptomic level, but it does not provide patient-tissue co-localization of WTAP and TRAF6 with PAS-positive/CD31-negative structures. In addition, prognosis was evaluated for WTAP expression rather than for a histologically defined VM endpoint, and no claim is made that TRAF6, the axis score, or the VM-related signature is an independent prognostic factor.

Nevertheless, the TCGA findings remain associative. Co-expression of WTAP and TRAF6 does not demonstrate that WTAP directly regulates TRAF6, and transcriptomic pathway scores cannot establish m6A modification or functional VM formation. The observed association between WTAP expression and overall survival was based on an unadjusted Kaplan-Meier analysis and should not be interpreted as evidence that WTAP is an independent prognostic factor. In addition, TCGA bulk RNA-sequencing data cannot resolve the cellular sources of WTAP, TRAF6, or the VM-related transcriptional signals. Validation in patient-derived HCC tissues, tissue microarrays, spatially resolved datasets, and independent clinical cohorts is therefore still required.

Several methodological limitations should also be acknowledged. The original MeRIP-qPCR experiment lacked IgG immunoprecipitation and transcript-level positive and negative controls; the IgG control in the added YTHDF1 RIP experiment does not address these separate MeRIP limitations. YTHDF1 involvement was supported by RNA immunoprecipitation and depletion, but methylation-dependent recognition, site-specific modification, direct translation efficiency, and reader-dependent mRNA decay were not established. A siRNA-resistant YTHDF1 rescue was not included in the displayed experiments. The co-culture assay did not distinguish tumor-cell-autonomous VM from endothelial-assisted network formation, and the xenograft study did not include vascular tracers or direct blood-flow measurements. The displayed PAS/CD31 panels were qualitative. No formal *a priori* power calculation was performed for the animal experiments, and the original histological assessment was not fully blinded. These limitations restrict the mechanistic and translational scope of the conclusions. Endogenous TRAF6 3′UTR editing was not performed, so the requirement for the candidate motifs at the native locus remains untested.

The downstream signaling relationship between TRAF6 and the vascular plasticity-associated phenotype also remains unresolved. Although TRAF6 is a known upstream regulator of NF-κB and the WTAP-TRAF6 axis score was positively associated with the TCGA TNFα/NF-κB signature, no NF-κB inhibition, p65 activation assay, or pathway epistasis experiment was performed. Similarly, the observed changes in VE-cadherin, MMP2, MMP9 and other markers are compatible with vascular plasticity and matrix-remodeling programs but do not define a specific TRAF6-to-VM effector cascade. NF-κB should therefore be regarded as a candidate downstream mechanism for future validation rather than an established dependency in the present study.

Model identity limits the disease-specific interpretation of these findings. HepG2 is hepatoblastoma-derived, and BEL-7402 is listed as a contaminated, HeLa-derived line (Cellosaurus BEL-7402, 2025; Cellosaurus Hep-G2, 2026). Thus, results in these models do not establish a mechanism specific to adult HCC. The Huh7 co-culture and construct-validation findings and the TCGA-LIHC associations provide complementary evidence, but they do not replace validation of the full mechanism in authenticated adult HCC models. The male-only xenograft design also does not address sex-dependent effects.

In summary, the present findings place TRAF6 within a WTAP-regulated program associated with cell viability, motility, invasion, vascular-like network support, and xenograft growth, while the TCGA-LIHC analysis provides complementary evidence that WTAP and TRAF6 co-expression is associated with vascular plasticity-related transcriptional programs in human HCC. WTAP expression was associated with increased m6A enrichment and stability of TRAF6 mRNA, and rescue experiments established a functional contribution of TRAF6. The added experiments implicate YTHDF1 in TRAF6 protein regulation but do not connect this reader directly to transcript stabilization. Further studies should map the relevant m6A sites and distinguish reader-dependent translation, protein turnover, and RNA stability, evaluate tumor-cell-only VM and vascular perfusion in orthotopic models, and validate the pathway in human HCC specimens.

## Conclusion

In summary, the present study suggests that WTAP promotes cell viability, motility, invasion, vascular-like network support, and xenograft growth, at least in part through TRAF6-dependent regulation associated with increased TRAF6 m6A enrichment and mRNA stability. WT/MUT reporter responses, YTHDF1-associated TRAF6 RNA recovery, and attenuation of TRAF6 protein expression after YTHDF1 depletion provide additional evidence implicating YTHDF1 in this regulatory pathway. These findings support WTAP-TRAF6 regulation of the examined cellular and vascular plasticity-associated phenotypes, accompanied by qualitative VM-like histology. The precise methylated sites, the contribution of YTHDF1 to translation versus other post-transcriptional processes, functional vascular perfusion, and clinical relevance remain to be established. Accordingly, the present data support a partial WTAP-TRAF6 regulatory axis associated with vascular plasticity-related phenotypes rather than a fully established site-specific WTAP-reader-TRAF6-NF-κB-VM pathway.

## Data Availability

The raw data supporting the conclusions of this article will be made available by the authors, without undue reservation.

## References

[B1] Cai Z. Liu R. Zhao L. Zhou L. Li Q. He H. (2026). WTAP-Mediated N6-methylation of TRAF6 facilitates polycystic ovary syndrome by inducing ferroptosis. Reprod. Sci. 33, 1646–1658. 10.1007/s43032-026-02121-8 42115577

[B2] Cellosaurus BEL-7402 (CVCL_5492) (2025). BEL-7402 (CVCL_5492). Available online at: https://www.cellosaurus.org/CVCL_5492 (Accessed 20 September 2026).

[B3] Cellosaurus Hep-G2 (CVCL_0027) (2026). Hep-G2 (CVCL_0027). Available online at: https://www.cellosaurus.org/CVCL_0027 (Accessed 20 September 2026).

[B4] Chang W. Feng K. Zhou P. Gong D. Wang K. Huang A. (2025). SPOP suppresses hepatocellular carcinoma growth and metastasis by ubiquitination and proteasomal degradation of TRAF6. Cancer Sci. 116, 1295–1307. 10.1111/cas.70025 39962908 PMC12044664

[B5] Chen Y. Peng C. Chen J. Chen D. Yang B. He B. (2019). WTAP facilitates progression of hepatocellular carcinoma via m6A-HuR-dependent epigenetic silencing of ETS1. Mol. Cancer 18, 127. 10.1186/s12943-019-1053-8 31438961 PMC6704583

[B6] Folberg R. Maniotis A. J. (2004). Vasculogenic mimicry. APMIS 112, 508–525. 10.1111/j.1600-0463.2004.apm11207-0810.x 15563313

[B7] Huang B. Xiao E. Huang M. (2015). MEK/ERK pathway is positively involved in hypoxia-induced vasculogenic mimicry formation in hepatocellular carcinoma which is regulated negatively by protein kinase A. Med. Oncol. 32, 408. 10.1007/s12032-014-0408-7 25487444

[B8] Li N. Luo L. Wei J. Liu Y. Haque N. Huang H. (2021). Identification of a new TRAF6 inhibitor for the treatment of hepatocellular carcinoma. Int. J. Biol. Macromol. 182, 910–920. 10.1016/j.ijbiomac.2021.04.081 33865893

[B9] Li X. Liu C. Zhang Z. Li X. Yao Z. Dong Y. (2023). Hepatocyte-specific wtap deficiency promotes hepatocellular carcinoma by activating GRB2-ERK depending on downregulation of proteasome-related genes. J. Biol. Chem. 299, 105301. 10.1016/j.jbc.2023.105301 37777158 PMC10630636

[B10] Li J. Ding X. Yan W. Liu K. Ye W. Wang H. (2024). Tumor-derived immunoglobulin-like transcript 4 promotes postoperative relapse via inducing vasculogenic mimicry through MAPK/ERK signaling in hepatocellular carcinoma. Am. J. Pathol. 194, 2398–2411. 10.1016/j.ajpath.2024.08.010 39233275

[B12] Liang L. Xu H. Dong Q. Qiu L. Lu L. Yang Q. (2022). WTAP is correlated with unfavorable prognosis, tumor cell proliferation, and immune infiltration in hepatocellular carcinoma. Front. Oncol. 12, 852000. 10.3389/fonc.2022.852000 35480109 PMC9035869

[B13] Liu J. Yue Y. Han D. Wang X. Fu Y. Zhang L. (2014). A METTL3-METTL14 complex mediates Mammalian nuclear RNA N6-adenosine methylation. Nat. Chem. Biol. 10, 93–95. 10.1038/nchembio.1432 24316715 PMC3911877

[B14] Llovet J. M. Kelley R. K. Villanueva A. Singal A. G. Pikarsky E. Roayaie S. (2021). Hepatocellular carcinoma. Nat. Rev. Dis. Prim. 7, 6. 10.1038/s41572-024-00500-6 33479224 PMC13537433

[B15] Maniotis A. J. Folberg R. Hess A. Seftor E. A. Gardner L. M. Pe'er J. (1999). Vascular channel formation by human melanoma cells *in vivo* and *in vitro:* vasculogenic mimicry. Am. J. Pathol. 155, 739–752. 10.1016/S0002-9440(10)65173-5 10487832 PMC1866899

[B16] Marques Dos Reis E. Vieira Berti F. (2022). Vasculogenic Mimicry-An overview. Methods Mol. Biol. 2514, 3–13. 10.1007/978-1-0716-2403-6_1 35771413

[B17] Morse M. A. Sun W. Kim R. He A. R. Abada P. B. Mynderse M. (2019). The role of angiogenesis in hepatocellular carcinoma. Clin. Cancer Res. 25, 912–920. 10.1158/1078-0432.CCR-18-1254 30274981

[B18] Ou H. Chen Z. Xiang L. Fang Y. Xu Y. Liu Q. (2019). Frizzled 2-induced epithelial-mesenchymal transition correlates with vasculogenic mimicry, stemness, and hippo signaling in hepatocellular carcinoma. Cancer Sci. 110, 1169–1182. 10.1111/cas.13949 30677195 PMC6447835

[B19] Ping X. L. Sun B. F. Wang L. Xiao W. Yang X. Wang W. J. (2014). Mammalian WTAP is a regulatory subunit of the RNA N6-methyladenosine methyltransferase. Cell Res. 24, 177–189. 10.1038/cr.2014.3 24407421 PMC3915904

[B20] Qiao K. Liu Y. Xu Z. Zhang H. Zhang H. Zhang C. (2021). RNA m6A methylation promotes the formation of vasculogenic mimicry in hepatocellular carcinoma *via* hippo pathway. Angiogenesis 24, 83–96. 10.1007/s10456-020-09744-8 32920668

[B21] Qin L. N. Zhang H. Li Q. Q. Wu T. Cheng S. B. Wang K. W. (2024). Vitamin D binding protein (VDBP) hijacks twist1 to inhibit vasculogenic mimicry in hepatocellular carcinoma. Theranostics 14, 436–450. 10.7150/thno.90322 38164156 PMC10750215

[B23] Wu H. Yang T. Y. Li Y. Ye W. L. Liu F. He X. S. (2020). Tumor necrosis factor receptor-associated factor 6 promotes hepatocarcinogenesis by interacting with histone deacetylase 3 to enhance c-Myc gene expression and protein stability. Hepatology 71, 148–163. 10.1002/hep.30801 31155734

[B24] Wu Z. Lin Y. Wei N. (2023). N6-methyladenosine-modified HOTAIRM1 promotes vasculogenic mimicry formation in glioma. Cancer Sci. 114, 129–141. 10.1111/cas.15578 36086906 PMC9807531

[B25] Yang Z. Sun B. Zhao X. Shao B. An J. Gu Q. (2015). Erythropoietin and erythropoietin receptor in hepatocellular carcinoma: correlation with vasculogenic mimicry and poor prognosis. Int. J. Clin. Exp. Pathol. 8, 4033–4043. 26097591 PMC4466978

[B26] Yao C. Wu S. Kong J. Sun Y. Bai Y. Zhu R. (2023). Angiogenesis in hepatocellular carcinoma: mechanisms and anti-angiogenic therapies. Cancer Biol. Med. 20, 25–43. 10.20892/j.issn.2095-3941.2022.0449 36647777 PMC9843448

[B27] Zhang S. Zhang D. Sun B. (2007). Vasculogenic mimicry: current status and future prospects. Cancer Lett. 254, 157–164. 10.1016/j.canlet.2006.12.036 17306454

[B28] Zhang Y. Che N. Wang S. Meng J. Zhao N. Han J. (2024a). Nrf2/ASPM axis regulated vasculogenic mimicry formation in hepatocellular carcinoma under hypoxia. J. Gastroenterol. 59, 941–957. 10.1007/s00535-024-02140-9 39097533

[B29] Zhang R. Yao Y. Gao H. Hu X. (2024b). Mechanisms of angiogenesis in tumour. Front. Oncol. 14, 1359069. 10.3389/fonc.2024.1359069 38590656 PMC10999665

[B30] Zong X. Xiao X. Shen B. Jiang Q. Wang H. Lu Z. (2021). The N6-methyladenosine RNA-Binding protein YTHDF1 modulates the translation of TRAF6 to mediate the intestinal immune response. Nucleic Acids Res. 49, 5537–5552. 10.1093/nar/gkab343 33999206 PMC8191762

